# Liquid–liquid phase separation couples MKRN2-mediated ubiquitination of CSDE1 with neurodevelopmental disorders

**DOI:** 10.3389/fncel.2026.1757304

**Published:** 2026-02-11

**Authors:** Zi Wang, Yaning Han, Peng Yang, Caiwei Jia, Chuanyin Li, Shilin Yuan, Pengfei Wei, Ronggui Hu

**Affiliations:** 1 National Center for Mental Disorders, Shanghai Mental Health Center, Brain Health Institute, Shanghai Jiao Tong University School of Medicine, Shanghai, China; 2 State Key Laboratory of Molecular Biology, Center for Excellence in Molecular Cell Science, Shanghai Institute of Biochemistry and Cell Biology, Chinese Academy of Sciences, Shanghai, China; 3 Department of Regeneration Science and Engineering, Institute for Frontier Life and Medical Sciences, Kyoto University, Kyoto, Japan; 4 Joint Laboratory of Guangdong-Hong Kong Universities for Vascular Homeostasis and Diseases, Department of Pharmacology, SUSTech Homeostatic Medicine Institute, School of Medicine, Southern University of Science and Technology, Shenzhen, China; 5 School of Life Science and Technology, ShanghaiTech University, Shanghai, China; 6 Department of Colorectal Surgery and Oncology (Key Laboratory of Cancer and Intervention, China National Ministry of Education), The Second Affiliated Hospital, Zhejiang University School of Medicine, Hangzhou, China; 7 Center for Medical Research and Innovation in Digestive System Tumors, Ministry of Education, Hangzhou, China; 8 State Key Laboratory of Digital Medicine, School of Biological Science and Medical Engineering, Southeast University, Nanjing, China; 9 Liangzhu Laboratory, State Key Laboratory of Brain-Machine Intelligence, MOE Frontier Science Center for Brain Science and Brain-Machine Integration, Zhejiang University, Hangzhou, China

**Keywords:** autism spectrum disorder, Csde1, liquid–liquid phase separation, MKRN2, ubiquitination

## Abstract

**Background:**

Makorin-2 (MKRN2) is an E3 ubiquitin ligase involved in multiple biological processes, yet its role in neurological disorders remains poorly understood. This study aims to elucidate how MKRN2 regulates the RNA-binding protein CSDE1—a molecule linked to autism-related genes—and to explore the functional implications of this interaction in neurodevelopment.

**Methods:**

Using mass-spectrometry screening, we identified CSDE1 as a direct substrate of MKRN2. Ubiquitination sites were validated through mutagenesis of conserved lysine residues. Liquid–liquid phase separation (LLPS) assays were performed in HEK293 and SH-SY5Y cells, and behavioral phenotypes were assessed in *Mkrn2*-knockout mice. Statistical analyses included appropriate tests for comparing ubiquitination levels, condensate formation, and social behavior outcomes.

**Results:**

MKRN2 mediates CSDE1 ubiquitination at four lysine residues (K81, K91, K208, K727). Deletion of *MKRN2* or mutation of these sites abolished ubiquitination. MKRN2 and CSDE1 formed co-localized condensates via LLPS, which was disrupted by functional impairment of either protein. *Mkrn2*-knockout mice exhibited sex-specific social abnormalities—increased sociability in males and social withdrawal in females—recapitulating autism-spectrum disorder (ASD) heterogeneity. We further identified *MARK1* and *HNRNPUL2*, ASD-associated mRNAs, as ubiquitination-dependent targets of CSDE1, linking aberrant condensate dynamics to synaptic plasticity deficits.

**Conclusion:**

Our study reveals an LLPS-coupled ubiquitination mechanism by which MKRN2 regulates CSDE1, providing a novel molecular pathway underlying neurodevelopmental disorders. These findings offer new insights for understanding and treating neurological diseases such as ASD.

## 1 Introduction

The Makorin RING zinc finger (*MKRN*) gene family, which includes *MKRN2*, encodes E3 ubiquitin ligases characterized by distinctive zinc finger motifs that play essential roles in maintaining protein homeostasis and supporting neuronal function (Shin et al., 2017; Zhang et al., 2000; Yang et al., 2008). MKRN2 is ubiquitously expressed across tissues and contains a C3HC4 RING domain essential for its ubiquitination activity. Dysregulation of MKRN2 has been associated with impairments in neurodevelopment (Gray et al., 2001; Cheung et al., 2010). Previous mass spectrometry analyses from our group have identified multiple substrates of MKRN2, among which CSDE1 (Cold Shock Domain-Containing E1, also known as UNR) emerges as a molecule of particular interest.

CSDE1 is a highly conserved RNA-binding protein that regulates mRNA stability and translation (Chang et al., 2004; Youn et al., 2018), influencing key neuronal processes such as neuronal migration (Kobayashi et al., 2013; Meng et al., 2023), synaptic plasticity (Guo et al., 2019; Zeng et al., 2016), and neurogenesis (Guo et al., 2020; Ju Lee et al., 2017; Jia et al., 2025). Notably, CSDE1 target transcripts are enriched for genes associated with autism spectrum disorder (ASD) (Guo et al., 2019; Xia et al., 2014), and its misregulation has been shown to lead to aberrant dendritic spine morphology and synaptic dysfunction (Gangfuss et al., 2021; Krenn et al., 2022). Since CSDE1 is a substrate of the E3 ligase MKRN2 and both proteins are implicated in neurodevelopmental processes, their interaction may represent a new regulatory node in neuronal function.

Liquid–liquid phase separation (LLPS) is a fundamental mechanism underlying the spatiotemporal organization of biomolecules within cells. LLPS serves as a key mechanism organizing synaptic structures and signaling complexes (Milovanovic et al., 2018; Zeng et al., 2016; Zeng et al., 2018; Chen et al., 2020; Gomez et al., 2021), yet its dysregulation is implicated in both neurodevelopmental (e.g., ASD, Rett syndrome) and neurodegenerative (e.g., ALS, Alzheimer’s, and Parkinson’s) disorders (Milovanovic et al., 2018; Zeng et al., 2016; Zeng et al., 2018; Shih et al., 2022; Mu et al., 2024; Zbinden et al., 2020; Polymenidou and Cleveland, 2011; Ray et al., 2020; Ambadipudi et al., 2017).

In this study, we propose that MKRN2-mediated ubiquitination of CSDE1 may be regulated through an LLPS-dependent mechanism. Since CSDE1 was reported as an ASD-risk gene (Guo et al., 2019; Gangfuss et al., 2021) and one of the core stress granule components (Youn et al., 2018; Guo et al., 2019; Yang et al., 2020), the phase separation properties of MKRN2 could provide spatial control over CSDE1 ubiquitination, thereby influencing its role in neurodevelopment. Disruption of this regulatory axis may contribute to synaptic abnormalities observed in ASD and related neuropsychiatric conditions.

Therefore, elucidating how MKRN2-mediated ubiquitination of CSDE1 is modulated by LLPS could uncover novel therapeutic targets for neurodevelopmental and neurodegenerative disorders. Our studies also aim to determine whether MKRN2’s phase separation capacity influences its ubiquitination activity toward CSDE1 and whether this interplay affects CSDE1’s RNA-binding functions or protein stability. Such mechanistic insights will deepen our understanding of the molecular pathways underlying neuronal pathogenesis and may open new avenues for intervention.

## 2 Materials and methods

### 2.1 Animals

*Mkrn2*^(−/−)^ (*Mkrn2* KO) mice (aged 2–3 months, FVB background) were bought from cyagen biosciences company (Suzhou, China). These mice were then backcrossed for 10 generations to our C57BL/6 J strain. This *Mkrn2* strain was maintained in C57 background. *Mkrn2* knockout mice were generated by sibmating *Mkrn2* heterozygotes. Genomic DNAs isolated from the tails were genotyped with indicated primers (Table 1) by PCR as described (Xu et al., 2018; Liu et al., 2024). Mice were housed in a specific-pathogen-free (SPF) facility with 12-h light/dark cycle and ad libitum access to food and water. Per cage was housed three to five mice by genotype. All behavioral experiments were performed during the light cycle. All animal studies were performed strictly in accordance with the instructions of the Institutional Animal Care and Use Committee (IACUC) at Shanghai Institute of Biochemistry and Cell Biology, CAS. Female and male mice were both used for experiments in this study, with C57BL/6 genetic background (SLAC, China).

**Table 1:** Primers for KO mice genotyping and constructs used in the research.

Gene name		Primers
MKRN2 KO mice genotyping		
Homozygotes:	Forward primer	5′-AATATGCTGAACCCCACAGTCTT-3′
	Reverse primer	5′-TACCAGCTATGGATTTCTGGACAC-3′
Wildtype allele:	Forward primer	5′-AATATGCTGAACCCCACAGTCTT-3′
	Reverse primer	5′-AAACATGTCAGGAAAGAAAACCCG-3′
Mutants and truncations		
pEGFP-C1-MKRN2	Forward primer	5′-GCATGGACGAGCTGTACAAGatgagcaccaagcagatcac-3′
	Reverse primer	5′-GATCAGTTATCTAGATCCGGTttagggttctgatgattccac-3′
pEGFP-MKRN2-truncated 1	Reverse primer	5′-CTGTACAAGTCCGGACTCAGATatgagcaccaagcagatcac-3′
	Forward primer	5′-GATCAGTTATCTAGATCCGGTcacctccccgtgcaggtagac-3′
pEGFP-MKRN2-truncated 2	Forward primer	5′-CTGTACAAGTCCGGACTCAGATatgtgtgaaatctgtaggctgcaag-3′
	Reverse primer	5′-GATCAGTTATCTAGATCCGGTcatctcgtgttcgaacgtcaac-3′
pEGFP-MKRN2-truncated 3	Forward primer	5′-CTGTACAAGTCCGGACTCAGATatggaaaaggcctttgccttccag-3′
	Reverse primer	5′-GATCAGTTATCTAGATCCGGTtgatatcacacggcattctggac-3′
pEGFP-MKRN2-truncated 4	Forward primer	5′-CTGTACAAGTCCGGACTCAGATatggagtttgtaattccaagtgtg-3′
	Reverse primer	5′-GATCAGTTATCTAGATCCGGTttagggttctgatgattccac-3′
pmCherry-C1-CSDE1	Forward primer	5′-GGCATGGACGAGCTGTACAAGatgagctttgatccaaacc-3′
	Reverse primer	5′-GATCAGTTATCTAGATCCGGTttagtcaatgacaccagcttg-3′
pmCherry-C1-CSDE1-truncated 1	Forward primer	5′-CTCAGATCTCGAGCTCAAGCTTCatgagctttgatccaaacc-3′
	Reverse primer	5′-GATCAGTTATCTAGATCCGGTtgtatttgacagaacttctatattgg-3′
pmCherry-C1-CSDE1-truncated 2	Forward primer	5′-CTCAGATCTCGAGCTCAAGCTTCatgtttcagttcactaatgaagc-3′
	Reverse primer	5′-GATCAGTTATCTAGATCCGGTtcgccaaacattacaggcgctg-3′
pmCherry-C1-CSDE1-truncated 3	Forward primer	5′-CTCAGATCTCGAGCTCAAGCTTCatggtctgtgagggccccaag-3′
	Reverse primer	5′-GATCAGTTATCTAGATCCGGTttagtcaatgacaccagcttg-3′
pGEX4T-1-MKRN2	Forward primer	5′-CCAAAATCGGATCTGGTTCCGCGTGGATCCatgagcaccaagcagatcac-3′
	Reverse primer	5′-GTCAGTCAGTCACGATGCGGCCGCTCGAGgggttctgatgattccactcc-3′
pGEX4T-1-MKRN2 truncated 1	Forward primer	5′-CCAAAATCGGATCTGGTTCCGCGTGGATCCatgagcaccaagcagatcac-3′
	Reverse primer	5′-GTCAGTCAGTCACGATGCGGCCGCTCGAGcacctccccgtgcaggtagac-3′
pGEX4T-1-MKRN2 truncated 2	Forward primer	5′-CCAAAATCGGATCTGGTTCCGCGTGGATCCatgtgtgaaatctgtaggctg-3′
	Reverse primer	5′-CGTCAGTCAGTCACGATGCGGCCGCTCGAGcatctcgtgttcgaacgtc-3′
pGEX4T-1-MKRN2 truncated 3	Forward primer	5′-CAAAATCGGATCTGGTTCCGCGTGGATCCatggaaaaggcc′tttgccttc-3′
	Reverse primer	5′-CAGTCAGTCACGATGCGGCCGCTCGAGtgatatcacacggcattctg-3′
pGEX4T-1-MKRN2 truncated 4	Forward primer	5′-CAAAATCGGATCTGGTTCCGCGTGGATCCatggagtttgtaattccaag-3′
	Reverse primer	5′-GTCAGTCAGTCACGATGCGGCCGCTCGAGgggttctgatgattccactcc-3′
pET-28a-CSDE1	Forward primer	5′-CTCAGTGGTGGTGGTGGTGGTGctcgaggtcaatgacaccagc-3′
	Reverse primer	5′-CTGGTGGACAGCAAATGGGTCGCGGATCCatgagctttgatcc-3′
pET-28a-CSDE1 truncated 1	Forward primer	5′-CTCAGTGGTGGTGGTGGTGGTGctcgagtgtatttgacagaac-3′
	Reverse primer	5′-GACAGCAAATGGGTCGCGGATCCatgagctttgatccaaacc-3′
pET-28a-CSDE1 truncated 2	Forward primer	5′-CTCAGTGGTGGTGGTGGTGGTGCTCGAGtcgccaaacattacaggcgc-3′
	Reverse primer	5′-GGTGGACAGCAAATGGGTCGCGGATCCATGtttcagttcactaatgaag-3′
pET-28a-CSDE1 truncated 3	Forward primer	5′-CTCAGTGGTGGTGGTGGTGGTGCTCGAGgtcaatgacaccagcttgacgg-3′
	Reverse primer	5′-CTGGTGGACAGCAAATGGGTCGCGGATCCatggtctgtgagggccccaag-3′
pmCherry-Csde1-K81R	Forward primer	5′-GACGGACTGGGAGACCCATTGCTGTTAAACTGGTGAAG-3′
	Reverse primer	5′-CAATGGGTCTCCCAGTCCGTCGGTCCGATGATACTTCAAATTC-3′
pmCherry-Csde1-K91R	Forward primer	5′-GGTGAAGATAAGACAAGAAATCCTCCCTGAAGAACGAATG-3′
	Reverse primer	5′-GATTTCTTGTCTTATCTTCACCAGTTTAACAGCAATGGGTTTCCC-3′
pmCherry-Csde1-K135R	Forward primer	5′-GATAACAATAGACATACTGGTGCTGTAAGTGCTCGCAAC-3′
	Reverse primer	5′-CCAGTATGTCTATTGTTATCAATTACAAAGTTTATTTTATCTCC-3′
pmCherry-Csde1-K208R	Forward primer	5′-CAGAAATGGTAGAGAAGTTGCAACAGATGTCAGACTATTGCCTC-3′
	Reverse primer	5′-GTTGCAACTTCTCTACCATTTCTGTCCTTGATTGTGAATTCCACATC-3′
pmCherry-Csde1-K246R	Forward primer	5′-GTACCCAGTAGAAACCAGAATGACCCATTGCCAGGACGCATC-3′
	Reverse primer	5′-CATTCTGGTTTCTACTGGGTACTTTTGGGATAACTTTGGTTACAG-3′
pmCherry-Csde1-K333R	Forward primer	5′-GTTTCATCAGGTGTGTGGATCGTGATGTTCGTATGTTCTTCC-3’
	Reverse primer	5′-GATCCACACACCTGATGAAACCAAAACCATCTCTCATGGCAG-3′
pmCherry-Csde1-K727R	Forward primer	5′-GTCAATCGCTTGAGGAATATCACTCTGGATGATGCCAGTGC-3′
	Reverse primer	5′-GATATTCCTCAAGCGATTGACCAACCGATCAGGTCGAGGAG-3′

### 2.2 Cell culture and transfection

The human embryonic kidney cell line HEK293T were purchased from the cell bank of Chinese Academy of Sciences (CAS). The human neuroblastoma cell line SHSY5Y was purchased from the American Type Culture Collection (ATCC, Manassas, United States). All cells were cultured in Dulbecco’s modified Eagle’s medium (DMEM, high glucose), supplemented with 10% fetal bovine serum (FBS) and penicillin/streptomycin (all from Gibco, United States) in a 37 °C humidified atmosphere of 5% CO2. Plasmids used in this study were transfected into cells using polyethylenimine (Sigma) according to the manufacturer’s instructions. Briefly, cells were seeded into 6-well-plates, transfections were done when cells to be 90% confluent. 3 μg plasmids DNA and 9 μL polyethylenimine were diluted in 100 μL Opti-MEM Medium (Gibco, United States) respectively, incubated at room temperature for 5 min, and then mixed together for 20 min before added to cells. 48 h after trasfection, cells were harvested and prepared for Immunoprecipitation.

HEK293 or SHSY5Y-based mono or double-allele knockout cell lines were generated by genome editing using the CRISPR/Cas9 system (Li et al., 2020; Komor et al., 2017; Hsu et al., 2013). Five sgRNAs targeting *MKRN2* was designed as previously reported (Li et al., 2020; Komor et al., 2017; Hsu et al., 2013) and three sgRNAs targeting CSDE1, as listed below (target sequences are lowercase):

1) *MKRN2*-sg1-Forward: 5′-CACCG catctgcaagtactaccaga-3′
2) *MKRN2*-sg1-Reverse: 5′-AAAC tctggtagtacttgcagatg C-3′
3) *MKRN2*-sg2-Forward: 5′-CACCG gtgcctattctcacatgact-3′
4) *MKRN2*-sg2-Reverse: 5′-AAAC agtcatgtgagaataggcac C-3′
5) *MKRN2*-sg5-Forward: 5′-CACCG attcgacccagagcagagga-3′
6) *MKRN2*-sg5-Reverse: 5′-AAAC tcctctgctctgggtcgaat C-3′
7) *CSDE1*-sg1-Forward: 5′-CACCG tatcatcggaccgacggact-3′
8) *CSDE1*-sg1-Reverse: 5′-AAAC agtccgtcggtccgatgata C-3′
9) *CSDE1*-sg3-Forward: 5′-CACCG gtcgagtatagcttgtccaa-3′
10) *CSDE1*-sg3-Forward: 5′-AAAC ttggacaagctatactcgac C-3′

Twenty-four hours after transfection, cells were screened by 2 μg/mL puromycin (Beyotime, China) for 7 days. Single-cell colonies were picked, amplified, and confirmed by immunoblotting analysis. We then extracted genomic DNAs, amplified the specific target sequences, and performed Sanger sequencing to verify the aimed edits in the genomes to established the stably transfected cell lines.

### 2.3 CSDE1 and MKRN2 intracellular phase diagrams

HEK293 or SHSY5Y control and *MKRN2* KO cells were transfected with EGFP*-MKRN2* or mCherry*-CSDE1* or GFP*-G3BP1* plasmids. After 24 h of expression, cells were digested and then spread on a 29 mm dish with 20 mm glass-bottom well. 48 h after transfection, cells were stressed with 100 mM sodium arsenite for 20–30 min at 37 °C before observation for Phase separation. Images were captured using Olympus SpinSR and Zeiss LSM900 confocal laser-scanning microscope with a 60x objective. Phase diagrams were constructed by measuring fluorescence intensity in each cell and assessing the presence of SGs using G3BP1 as a marker (Yang et al., 2020), using Fiji software.

### 2.4 Liquid–liquid phase separation

*In vitro* LLPS experiments were performed at room temperature unless otherwise indicated. Purified EGFP-MKRN2 and mcherry-CSDE1 proteins were diluted with a phase separation buffer containing 25 mM Tris–HCl (pH = 7.4), 150 mM KCl, 2.5% Glycerol, 2% PEG20000, and 0.5 mM DTT, and the mixtures were incubated for 5 min to induce phase separation (Alberti et al., 2018; Liu et al., 2024). LLPS of CSDE1 and MKRN2 were induced by addition of indicated concentrations of PEG20000 or mRNAs. The samples were mixed in a 29 mm dish with 10 mm glass bottom well right before observation. Proteins were diluted to various salt (50–600 mM KCl) and protein (300–1600 nΜ) concentrations and observed using a Zeiss LSM900 confocal laser-scanning microscope equipped with a x2.5 or x20 objective. All imaged were captured within 5 min after LLPS induction. For LLPS experiments involving mRNA addition, total RNA was first isolated from HEK293 cells using TRNzol Universal RNA Reagent (Yeasen, China). The isolated RNA was then reverse-transcribed into cDNA. Selected target mRNAs, identified through prior RIP-seq analysis, were synthesized via *in vitro* transcription from the resulting cDNA.

Protein and mRNA concentrations were determined by measuring absorbance at 280 nm using a Nano-300 spectrophotometer (Allsheng, China) and Nanodrop (Thermo Fisher Scientific).

### 2.5 FRAP analysis

FRAP analysis was performed on the Olympus SpinSR confocal laser-scanning microscope. GFP or mcherry signals in regions of interest (ROI) were fully photobleached by using a 405-nm laser. Fluorescence intensity of ROI between pre-bleached and at the start of recovery after bleaching was recorded by microscope. FRAP data were analyzed using GraphPad Prism 9.

### 2.6 Plasmids construction

The plasmids containing *CSDE1* and *MKRN2* were amplified from the cDNA of human HEK293T cells using specific primers in Table 1, and inserted into pCDNA3.0, pET28a or pGEX4T-1 vectors that digested with BamH I or Xho I, and recombination with amplified fragments using the ClonExpress II One Step Cloning Kit (Vazyme, China) according to the manufacturer’s instructions. Five sgRNAs for *MKRN2* were synthesized as oligos (Tingke, Shanghai, China), annealed and inserted into the lentiCRISPR v2 vector, transformation into Stbl3 bacteria, and all constructs were confirmed by sequencing.

### 2.7 Recombinant protein purification

GST tagged CSDE1 or His6- tagged MKRN2 were expressed in BL21 *E. coli* cells. After isopropyl-*β*-d-thiogalactopyranoside (IPTG, Sangon, China) induction, *E. coli* cells were pelleted by centrifuge, lysed in PBS buffer and incubated with glutathione or Ni^2+^ NTA agarose beads (Smart-Lifesciences and NanoMicro, China) to enrich the GST − or His6- tagged proteins, followed by elution with 20 mM reduced L-glutathione or 400 mM imidazole (Sangon) that dissolved in PBS buffer, and then dialysis in PBS buffer supplemented with final concentration of 25% glycerol before being aliquoted and preserved at −80 °C. The fluorescent proteins for LLPS followed the same procedures.

### 2.8 Reconstituting the *E. coli* ubiquitination system and mass spectrometry analysis to map ubiquitylation modification sites on substrates

*E1*(*UBA1*), *E2* (*UBCH5A*) and HA-*UB* were added to the first multiple cloning site (MCS) of the PACYC vector and *MKRN2* was added to the second multiple cloning site to construct the plasmid pACYC-HA-*UB*- *UBA1*—*UBCH5A* -*MKRN2*, which was co-transformed with pET28a-*CSDE1*-His6 in BL21 *E. coli* competent cells and screened with ampicillin plus chloramphenicol antibiotics. After induction with IPTG, the cells were lysed with 8 M urea lysis buffer (50 mM Tris-Cl, 50 mM Na_2_HPO_4_, 300 mM NaCl, 8 M urea, 0.5% NP-40 and 20 mM imidazole, pH 8.0) and then incubated with Ni-NTA agarose beads for 4 h at room temperature. The immunoprecipitates were washed three times with 8 M urea lysis buffer and denatured in 2x SDS protein loading buffer at 100 °C for 10 min before immunoblotting analysis. The CSDE1 proteins recovered from the *E. coli* ubiquitination system were subjected to mass spectrometry analysis for ubiquitination site mapping as previously described (Xu et al., 2018).

Procedures for MS analysis were as previously described (Xu et al., 2018). In brief, the protein pellet was concentrated in 8 M urea, 100 mM Tris-Cl (pH 8.5), followed by TCEP reduction, NEM alkylation, and trypsin digestion. Peptides were separated and analyzed by the EASY-nLC system (Thermo Fisher) and the Q Exactive mass spectrometer (Thermo Fisher), respectively. Ubiquitylation modification sites were determined with Thermo Proteome Discoverer 2.1 (Thermo Fisher) and searched in Uniprot Human database.^1^

### 2.9 GST pull-down assay

Purified GST or GST-MKRN2 (30 μg), CSDE1- His6 proteins (30 μg) and Glutathione Sepharose4B (Sangon) were incubated at 4 °C overnight in 800 μL GST pull-down buffer (20 mM Tris–HCl, 100 mM NaCl, 1 mM EDTA, 5 mM MgCl2, 1 mM DTT, 0.5%NP- 40, 10 μg/mL of BSA, pH 7.4). These beads are then pelletized and washed three times with a pull-down buffer. The recovered immunoprecipitates were boiled in 2xSDS-PAGE protein loading buffer for 10 min, and then subjected to immunoblotting analysis with the indicated antibodies (Jia et al., 2020).

### 2.10 Immunoprecipitation and immunoblotting

Exogenous or endogenous protein-expressing cells were lysed in IP buffer (50 mM Tris-Cl, pH 7.5, 150 mM NaCl, 5 mM EDTA, 1% NP-40, 0.1% SDS, 0.5% Sodium deoxychlate) and CO-IP buffer (50 mM Tris–HCl, pH7.5, 150 mM NaCl, 5 mM EDTA and 1% NP-40) supplemented with protease inhibitor cocktail (LabEAD) (Li et al., 2020). Resuspended cells were lysed using a probe sonicator with 20% amplitude, 1 s pulse-on/2 s pulse-off cycles with total duration ≥1 min on ice and centrifuged at 15,000 rpm for 10 min at 4 °C; the supernatants were collected, and then incubated with specific antibodies or anti-Flag M2 affinity gels (Sigma, United States) at 4 °C overnight. The beads are pelletized and washed 3 times with IP buffer. The antibodies used were as follows: normal rabbit IgG (1:5,000 dilution, 2729S, Cell signaling), anti-MKRN2 (1:2,000, A12219, ABclonal), and anti-CSDE1 (1:2,000 dilution, 13319-1-AP, Proteintech). The immunoprecipitates were enriched and denatured at 99 °C for 10 min in 2xSDS-PAGE loading buffer. The immunoprecipitates and inputs were subjected to SDS-PAGE and then were transferred to PVDF membranes (Bio-Rad, United States). The membranes were blocked with 10% skimmed milk at RT for 1 h, and were immunoblotted with the specified antibodies as follows: anti-MKRN2, anti-CSDE1 and anti-Flag (1:1,000 dilution, 20543-1-AP, Proteintech), anti-Myc (1:1,000 dilution, 16286-1-AP, Proteintech), anti-HA (1:1,000 dilution, HRP-81290, Proteintech), anti-His (1:2000 dilution, 10001-0-AP, Proteintech), anti-GST (1:10,000 dilution, HRP-66001, Proteintech), and anti-GAPDH (1:5,000 dilution, 60004-1-Ig, Proteintech). Then the membranes were incubated with HRP-conjugated goat anti-mouse IgG (1:5,000 dilution; SA00001-1, Proteintech) or goat anti-rabbit IgG (1:5,000 dilution; SA00001-2, Proteintech) secondary antibodies for 1 h at room temperature. The protein–antibody complex was detected by a chemiluminescence detection system via an ECL chemiluminescence detection kit, and the signals were visualized using a Tanon 5,200 Imaging System (Tanon, Shanghai, China).

### 2.11 RNA immunoprecipitation sequencing (RIP-seq) experiments

RNA immunoprecipitation was performed as previously described (Keene et al., 2006) using *MKRN2* knockout SH-SY5Y cells. Briefly, cells transfected with Flag, *CSDE1*-Flag, and *MKRN2*-Myc plasmids were collected after 48 h, and the cells pellet were resuspended with an approximately equal volume of polysome lysis buffer[100 mM KCl, 5 mM MgCl_2_, 10 mM HEPES (pH 7.0), 0.5% NP40, 1 mM DTT, 100 units/mL RNase inhibitor (Vazyme), 400 μM vanadyl ribonucleoside complexes (VRC, NEB, United States) and Protease inhibitor cocktail (1:100, Roche)]. Incubated on ice for 10 min, centrifuged at 15,000 g for 15 min to clear lysate of large particles. Anti-FLAG M2 Affinity gels (Sigma, United States) were washed with NT2 buffer (50 mM Tris–HCl, 150 mM NaCl, 1 mM MgCl2, 0.05% NP-40 pH 7.4) before using for immunoprecipitation. 50 μL of pre-cleared supernatant lysate were saved as the total cellular mRNA (input), and the rest of RNA-protein complexes were immunoprecipitated with anti-FLAG M2 Affinity gels (Sigma, United States) in 850 μL of NT2 buffer supplemented with 100 units/mL RNase inhibitor, 400 μM VCR, 10 μL of 100 mM DTT and 20 mM EDTA, and incubated for 4 h at 4 °C with tumbling end over end. Beads were washed with 1.0 mL of ice-cold NT2 buffer for five times supplemented with 0.5 M urea and 0.1% SDS, and then resuspended the beads in 100 μL of NT2 buffer supplemented with 30 μg of proteinase K (Roche) to release the RNP components. RNAs were extracted using Trizol (Invitrogen), and subjected to RIP-seq analysis.

### 2.12 Quantitative real-time PCR

Total RNAs were extracted from cells using a Trizol reagent (Tiangen, China). Complementary DNA (cDNA) was synthesized using HiScript III RT SuperMix for qPCR (+gDNA wiper) (Vazyme #R323). Quantitative PCR (qPCR) assay was performed to determine the relative abundances of selected mRNAs using specific primers in Table 2, stained by SYBR Green (Tiangen, China) on the Roche LightCycler 480 II (384 well) real-time PCR system. The relative abundances of mRNAs were normalized to that of ATP2A1 mRNAs, using the 2^−∆∆Ct^ method as previously described (Xu et al., 2018). All experiments were repeated three times.

**Table 2:** Primers of RIP-seq analysis selected mRNAs for quantitative real-time PCR.

Gen name		Primers
EIF3G	Forward primer	CGCTGCCCCTACAAGGATAC
	Reverse primer	GCGGCACATACTTCCCTGTC
HACE1	Forward primer	AGTTGCCCGAGGATAATGAAAC
	Reverse primer	TCCACCGATCCACAATTTGCT
HNRNPUL2	Forward primer	CGAGAGGAGGCTTACCACAG
	Reverse primer	CGTGTCCAGGTTCACAAGAGT
KANK1	Forward primer	TCGAGGAAAAAGGTTGACAAAGC
	Reverse primer	TCCACCAGGTCCATGTGACT
MARK1	Forward primer	CCCCGGTGTAGAAACTCCATT
	Reverse primer	AGAACGTGTCTTGCCAATTTGA
PAX6	Forward primer	TGGGCAGGTATTACGAGACTG
	Reverse primer	ACTCCCGCTTATACTGGGCTA
RIMS1	Forward primer	TGGAAGTCATTAGAGCACGAAGC
	Reverse primer	CCCAGACAATCACCTGAAGAACT
SCN8A	Forward primer	CCTTTCACCCCTGAGTCACTG
	Reverse primer	AGGTCGCTGTTTGGCTTGG
SDC2	Forward primer	TTGACAACAGCTCCATTGAAGAA
	Reverse primer	CAGCTCTGGACTCTCTACATCC
SLC9A6	Forward primer	GAGGAGATCGTGTCCGAGAAG
	Reverse primer	GCCAGATTGTGAGAATGGTGAG
TCF4	Forward primer	TGCAAAGCCGAATTGAAGATCG
	Reverse primer	AGAAGGTCCAATGATTCCATGC
KDM4C	Forward primer	GATGAATGGAACATAGCTCGCC
	Reverse primer	GGTGTGCCATGCAAACGTG

Cross-referencing RIP-seq data with the SFARI autism gene database yielded 117 overlapped ASD genes. Eleven of selected mRNAs were checked with quantitative PCR (qPCR).

### 2.13 Mouse behavior test

#### 2.13.1 Self-groomming test

The Self-grooming test was performed as previously described (Wang et al., 2020). The mice used for the self-grooming test were firstly habituated in a cleaning chamber covered with beddings for 10 min. After the habituation, the time of mice spent in grooming was recorded within 10-min intervals, using stop-watches by two observers who were blinded to the groupings of mice. All instances of face- wiping, head and ears scratching/rubbing, and full-body grooming were counted as grooming behavior.

#### 2.13.2 Open field test

Locomotor activity was evaluated in an acrylic box (40 cm × 40 cm × 40 cm, Med Associates) and videotaped by an overhead camera. Mice, grouped in a blinded manner, were placed in the box and allowed to explore for 10 min. The time spent in center (a 20 cm × 20 cm area in the center of the bottom) and distances traveled in center compared with total traveling distances were measured using Ethovision automated tracking software (Noldus).

#### 2.13.3 Three-chamber social test

The three-chamber social test was performed according to previously reported protocols (Rein et al., 2020; Yang et al., 2022). In brief, a transparent acrylic box (60 cm × 40 cm × 20 cm) was equally divided into three chambers with removable doors in each partition. Two wire cups were placed with the opening down in the left and right chambers. Two days prior to the testing, two age- and gender-matched C57BL/6 mice, not littermates, were placed under both wire cups as stranger mice, and habituated for 1 h per day. Test mice, grouped in a blinded manner, were habituated to the test room for 1 h before the start of behavior tests.

In the sociability test phase, a stranger mouse (stranger I) and an inanimate object were placed into the right and left cages, randomly. The test mouse was allowed to explore all three chambers freely for 10 min and the amount of time spent in each chamber was recorded. Then the test mouse was asked to spend an extra 5 min in the stranger I chamber to get more familiar with stranger I before the next phase.

In the social novelty test phase, the inanimate object was replaced with a novel mouse (stranger II). Similarly, the test animal was allowed to freely explore all three sections of the apparatus for 10 min and the amount of time spent in each chamber was recorded. The sociability preference index = (time spent in stranger I chamber-time spent in object chamber)/(total time in the two chambers); social novelty preference index = (time spent in stranger II chamber-time spent in stranger I chamber)/(total time in the two chambers).

#### 2.13.4 Rotarod test

The motor coordination and balance ability of blindly grouped mice were examined using a Rotamex rotarod apparatus (Columnbus Instruments). In a single day, the mice were tested on three trials, with each trial lasting 10 min with the rod rotation accelerated from 4 to 40 rpm. The intervals permitting rest between each trial were about 30 min. The time of mouse falling down the ROD was automatically recorded by the infrared detection system and results of the last trial were compared.

#### 2.13.5 3D-free social interactions: the social behavior atlas

The high-resolution 3D free social interaction assays was collaborated with the Hu Ji Lab from ShanghaiTech University and the Wei Pengfei Lab from Shenzhen Institute of Advanced Technology using *Mkrn2* KO mice. The Hu Ji Lab captured 3D social interactions using multi-angle high-speed cameras, and the Wei Pengfei Lab processed and quantified the behavioral data using DeepLabCut-based 3D pose estimation and custom behavioral classifiers as previously described (Han et al., 2023; Huang et al., 2021).

### 2.14 Statistical analysis

All results have been obtained from at least three independent experiments, data of experiments are expressed as mean ± standard error of the mean (SEM) and analyzed by two-tailed unpaired *t*-test and one-way ANOVA with Tukey’s *post-hoc* test using GraphPad Prism 9. (GraphPad Software Inc., United States). ^∗^*p* < 0.05 was considered to be significantly different, ^∗∗^*p* < 0.01 was considered to be very significantly different, ∗∗∗*p* < 0.001 was considered to be highly significantly different, and ∗∗∗∗*p* < 0.0001 was considered to be extremely significantly different.

## 3 Results

### 3.1 MKRN2 interacts with, and ubiquitinates CSDE1

**Figure 1:**
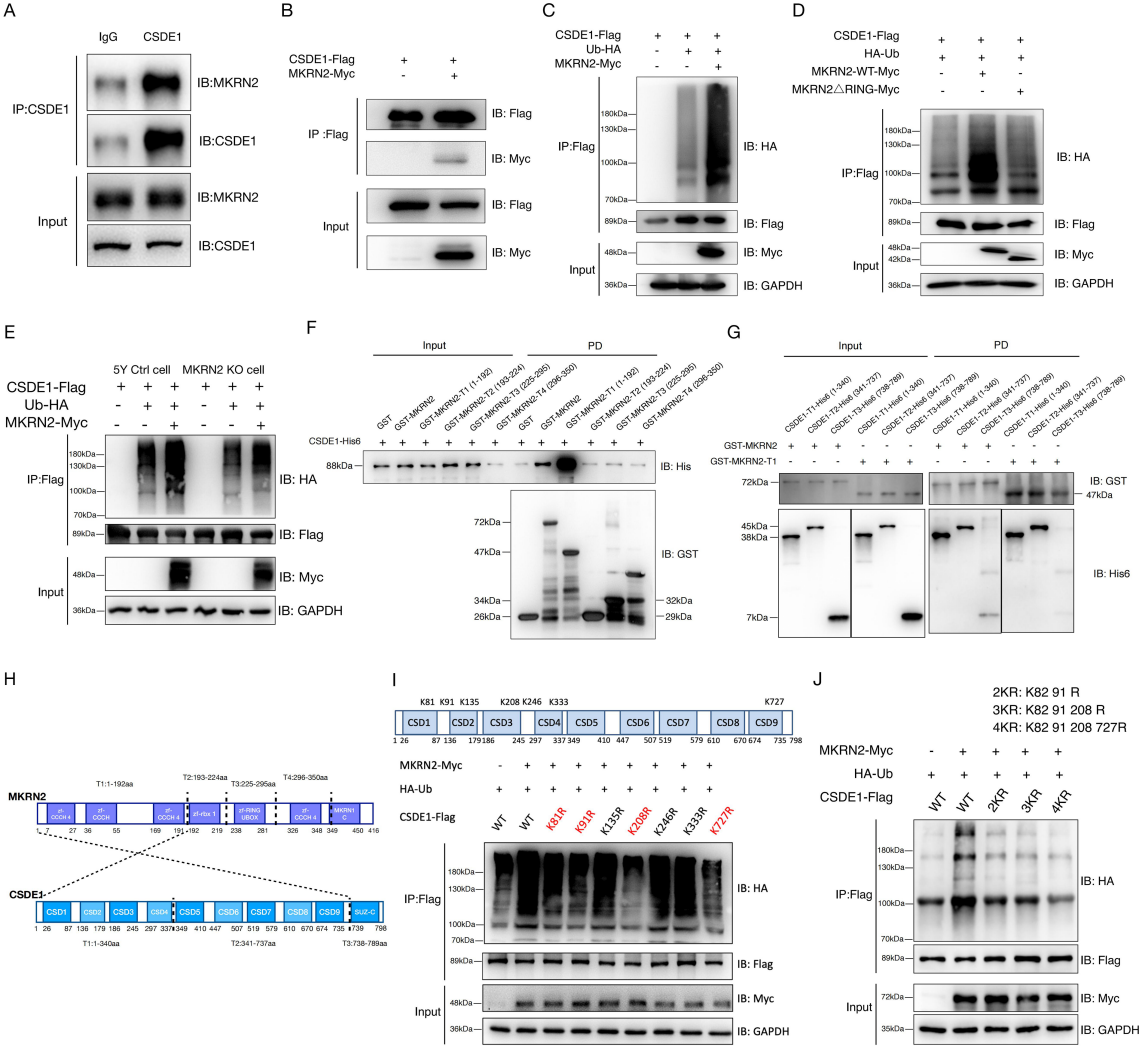
Human MKRN2 interacts with CSDE1 *in vitro* and *in vivo*. **(A)** Endogenous MKRN2 interacted with CSDE1 in SHSY5Y cells. The lysates of SHSY5Y cells were immunoprecipitated with anti-CSDE1 antibody and detected by immunoblotting. **(B)** MKRN2 interacted with CSDE1 in SHSY5Y cells. SHSY5Y cells were transfected with MKRN2-Myc and CSDE1-Flag and immunoprecipitated using an anti-Flag affinity gel after 48 h, followed by immunoblotting with the indicated antibodies. **(C)** CSDE1-Flag was ubiquitinated by MKRN2-Myc in HEK293T cells. Cells were co-transfected with MKRN2-Myc, HA-Ub, and CSDE1-Flag as indicated. **(D)** The RING domain of MKRN2 is essential for the ubiquitination of CSDE1. HEK293T cells were co-transfected with CSDE1-Flag, HA-Ub, MKRN2-Myc, or MKRN2 (△RING)-Myc as indicated. **(E)** CSDE1 ubiquitination is reduced in *MKRN2* KO cells compared to wild-type SHSY5Y cells. Reintroduced MKRN2 can rescue CSDE1 ubiquitination. *MKRN2* KO or wild-type SHSY5Y cells were co-transfected with HA-Ub, CSDE1-Flag, and/or MKRN2-Myc as indicated. **(F)** The direct interaction of MKRN2 with CSDE1 was detected by the GST pull-down assay. The N-terminal C3H zinc finger region of MKRN2 is primarily responsible for interacting with CSDE1. Reconstituted GST-tagged MKRN2 and His-tagged CSDE1 were purified, and the GST pull-down assay was performed and detected by immunoblotting. PD: GST pull-down. **(G,H)** Reciprocal binding experiments using truncated versions of CSDE1 and MKRN2, the N-terminal cold shock domains of CSDE1 mediate the strongest interaction with the N-terminal C3H zinc finger region of MKRN2. **(I)** Schematic distribution of the 7 sites (Lys residues) for MKRN2-mediated ubiquitination on human CSDE1. CSDE1 proteins were recovered from the *in vitro* ubiquitination assay and subjected to mass spectrum analysis to map the ubiquitination sites. Four Lys residues (81, 91, 208, and 727, shown in red) turned out to be the major sites for MKRN2-mediated ubiquitination of CSDE1 in HEK293T cells. **(J)** 2KR (81, 91), 3KR (81, 91, and 208), 4KR (81, 91, 208, and 727), the mutant simultaneously bearing K-to-R mutations in all the four Lys residues in CSDE1.

To identify potential substrates of MKRN2, we performed coimmunoprecipitation (Co-IP) followed by mass spectrometry and identified CSDE1 as a candidate substrate. Co-IP assays confirmed that both endogenous and exogenously expressed MKRN2 interact with CSDE1 in SH-SY5Y cells (Figures 1A,B). Fluorescence colocalization analysis further demonstrated strong cytoplasmic co-localization of MKRN2 and CSDE1 (Supplementary Figure 1A).

To determine whether MKRN2 mediates the ubiquitination of CSDE1, we co-expressed Myc-MKRN2 and Flag-CSDE1 in HEK293T cells. Co-IP analysis confirmed their interaction and revealed that MKRN2 promotes CSDE1 ubiquitination (Figure 1C). Deletion of the RING finger domain in MKRN2 significantly impaired its ability to ubiquitinate CSDE1, both in HEK293T cells and in a reconstituted *E. coli* ubiquitination system (Figure 1D; Supplementary Figure 1B), underscoring the essential role of this domain. Notably, CSDE1 ubiquitination was largely dependent on MKRN2, with minimal contribution from E1 or E2 enzymes alone (Supplementary Figure 1B).

To further investigate the functional role of MKRN2 in CSDE1 ubiquitination, we generated *MKRN2* knockout (KO) cell lines in both SH-SY5Y and HEK293 cells (Supplementary Figures 1C,D). Immunoprecipitation analyses revealed a marked reduction in CSDE1 ubiquitination in *MKRN2*-KO cells, accompanied by a modest increase in CSDE1 protein levels (Figure 1E; Supplementary Figure 1E). Re-expression of MKRN2 in KO cells partially restored CSDE1 ubiquitination, confirming that MKRN2 positively regulates this process. The residual ubiquitination observed in *MKRN2*-deficient cells suggests the involvement of additional E3 ligases or regulatory mechanisms.

To determine whether the MKRN2–CSDE1 interaction is direct, we performed *in vitro* GST pull-down assays using recombinant GST-MKRN2 and CSDE1-His₆ proteins. These assays confirmed direct binding between MKRN2 and CSDE1, independent of other cellular factors (Supplementary Figure 1F).

MKRN2 contains four conserved C3H zinc fingers, a Cys-His motif, and a C3HC4 RING domain, and has been implicated as a putative ribonucleoprotein (William et al., 2010). CSDE1 comprises five cold shock domains (CSD1–5) interspersed with unstructured linker regions and is known to function as an RNA-binding protein (Guo et al., 2019; Gangfuss et al., 2021). To map the domains responsible for MKRN2-mediated ubiquitination of CSDE1, we generated a series of truncated mutants for both proteins (Supplementary Figure 1G): four MKRN2 truncations (GST-MKRN2-T1 to T4) and three CSDE1 truncations (CSDE1-His₆-T1 to T3). GST pull-down assays showed that GST-MKRN2-T1, which contains the first three C3H zinc fingers, exhibited strong binding to CSDE1 (Figure 1F), indicating that the N-terminal C3H region of MKRN2 is primarily responsible for the interaction. Notably, in some experimental replicates including the representative data shown, the T4 fragment of MKRN2 also demonstrated a detectable interaction with full-length CSDE1. However, this interaction proved less consistent and robust compared to that mediated by the T1 fragment, and it was not reproducibly observed in subsequent domain-mapping assays using isolated truncations.

Reciprocal binding assays using truncated CSDE1 constructs revealed that the N-terminal cold shock domains (CSD1–5) of CSDE1 are critical for binding to the C3H zinc finger region of MKRN2 (Figures 1G,H). Collectively, these data demonstrate that the interaction between MKRN2 and CSDE1 is mediated by the N-terminal C3H zinc fingers of MKRN2 and the N-terminal cold shock domains of CSDE1, and that this interaction is essential for MKRN2-dependent ubiquitination of CSDE1.

### 3.2 MKRN2 mediates the ubiquitination of CSDE1 at multiple sites

To define how MKRN2 governs CSDE1 through ubiquitination, we conducted high-resolution mass-spectrometry mapping of CSDE1 ubiquitin acceptor sites. Seven ubiquitinated lysine (K) were confidently identified (Table 3; Figure 1I). To pinpoint the residues preferentially targeted by MKRN2, we systematically replaced each lysine with arginine (K → R) and monitored ubiquitination by co-IP. Mutation of four residues-K81, K91, K208, and K727-produced the strongest reduction in CSDE1 ubiquitination (Figure 1I). Stepwise combination of these mutations (double K81/91R, triple K81/91/208R and quadruple K81/91/208/727R) led to a progressive loss of ubiquitination that plateaued with the quadruple mutant (Figure 1J), establishing K81, K91, K208, and K727 as the principal MKRN2-directed ubiquitination sites on CSDE1.

**Table 3:** Mass spectrum analysis of CSDE1 ubiquitination sites.

Protein group	3	3	3	3	3	3	3	3	3	3
Protein ID	87	87	87	87	87	87	87	87	87	87
Protein accession	O75534|CSDE1_HUMAN	O75534|CSDE1_HUMAN	O75534|CSDE1_HUMAN	O75534|CSDE1_HUMAN	O75534|CSDE1_HUMAN	O75534|CSDE1_HUMAN	O75534|CSDE1_HUMAN	O75534|CSDE1_HUMAN	O75534|CSDE1_HUMAN	O75534|CSDE1_HUMAN
Peptide	K. INFVIDNNK(+114.04)HTGAVSAR. N	R. LK(+114.04)NITLDDASAPR. L	K. VPS(+114.04)KNQNDPLPGR. I	K. S(+114.04)KVTLLEGDHVR. F	R. DGFGFIK(+114.04)C(+125.05)VDR. D	K. IK(+114.04)QEILPEER. M	R. NGK(+114.04)EVATDVR. L	K. LPKGT(+114.04)VSFHSHSDHR. F	R. TGK(+114.04)PIAVK. L	I. NFVIDNNK(+114.04)HTGAVSAR. N
Unique	Y	Y	Y	Y	Y	Y	Y	Y	Y	Y
-10lgP	48.19	40.32	37.66	37.45	37.38	35.93	28.99	28.28	22.97	18.51
Mass	1969.0129	1526.8052	1534.7852	1466.7841	1494.6925	1367.7408	1201.6051	1817.8921	926.5549	1855.9288
Length	17	13	13	12	11	10	10	15	8	16
ppm	5.2	5.3	3.8	2	1.4	2.5	2	3.5	2.3	17.2
m/z	657.3483	764.4139	512.6042	367.704	499.2388	684.8794	601.811	455.4819	309.8596	619.6609
z	3	2	3	4	3	2	2	4	3	3
RT	36.27	35.76	18.76	27.84	72.55	28.8	11.12	13.1	11.72	40.09
Area sample 3	3.27E+06	4.32E+07	1.80E+06	1.76E+05	2.18E+05	1.65E+07	3.67E+05	1.40E+05	1.07E+06	5.21E+05
Fraction	3	3	3	3	3	3	3	3	3	3
Scan	13,544	13,335	6,245	10,040	28,806	10,439	3,095	3,903	3,338	15,157
Source file	huronggui103.raw	huronggui103.raw	huronggui103.raw	huronggui103.raw	huronggui103.raw	huronggui103.raw	huronggui103.raw	huronggui103.raw	huronggui103.raw	huronggui103.raw
#Spec	2	2	2	1	1	2	1	1	1	1
#Spec sample 3	2	2	2	1	1	2	1	1	1	1
Start	158	757	274	306	358	90	237	413	79	159
End	174	769	286	317	368	99	246	427	86	174
PTM	Ubiquitin	Ubiquitin	Ubiquitin	Ubiquitin	Ubiquitin; N-ethylmaleimide on cysteines	Ubiquitin	Ubiquitin	Ubiquitin	Ubiquitin	Ubiquitin
AScore	K9: Ubiquitin:26.31	K2: Ubiquitin:72.33	S3: Ubiquitin:0.00	S1: Ubiquitin:0.00	K7: Ubiquitin:1,000.00; C8: N-ethylmaleimide on cysteines:1000.00	K2: Ubiquitin:1000.00	K3: Ubiquitin:67.69	T5: Ubiquitin:0.00	K3: Ubiquitin:6.59	K8: Ubiquitin:32.28

The high-resolution mass-spectrometry mapping of CSDE1 ubiquitin acceptor sites. Seven ubiquitinated lysine (K) were identified.

### 3.3 The formation of CSDE1 and MKRN2 condensates under sodium arsenite treatment

CSDE1 is an RNA-binding protein that couples translation to mRNA turnover (Chang et al., 2004) and serves as one of seven core scaffolds of the stress-granule (SG) network, indispensable for efficient SG assembly (Youn et al., 2018; Guo et al., 2019). Emerging evidence shows that liquid–liquid phase separation (LLPS) drives the biogenesis of membrane-less organelles and is particularly important for synaptic organization (Chen et al., 2020; Gomez et al., 2021). Conversely, pathological LLPS is increasingly implicated in neurodevelopmental and neurodegenerative diseases (Milovanovic et al., 2018; Zeng et al., 2016; Zeng et al., 2018; Shih et al., 2022; Mu et al., 2024; Zbinden et al., 2020; Ambadipudi et al., 2017; Ray et al., 2020; Ambadipudi et al., 2017). These considerations prompted us to interrogate the phase-separation behavior of CSDE1 and its E3 ligase MKRN2.

**Figure 2:**
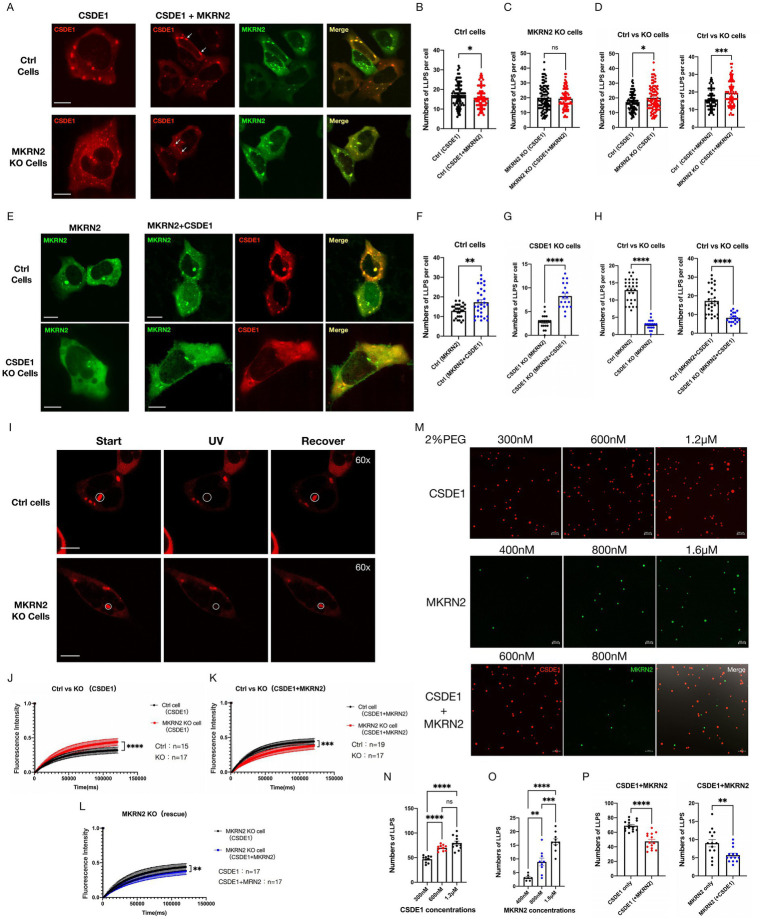
Numbers of SG formation relies on MKRN2 and CSDE1 interactions *in vivo* and *in vitro*. **(A)** CSDE1 and MKRN2 are recruited to SGs under oxidative stress. HEK293 WT and *MKRN2* knockout cells treated with sodium arsenite (500 μM, 30 min) were overexpressed with mCherry-*CSDE1*(red) and EGFP-*MKRN2* (green). After 48 h, images were acquired using the Olympus spinSR confocal microscope. Scale bar: 10 μm. **(B–D)** Quantification of CSDE1 condensate number per cell, **(B)** overexpression of MKRN2 inhibits SG formation, **(C)** supplementation of MKRN2 partially rescued the phenotype caused by *MKRN2* deficiency, **(D)** *MKRN2* deficiency increase SG formation, show as means ± SEM, *p < 0.05, **p < 0.01, ns: not significant. **(E)** Fewer CSDE1 and MKRN2 are recruited and co-localized to SGs under oxidative stress. HEK293 Control and *CSDE1* knockout cells treated with sodium arsenite (500 μM, 30 min) were overexpressed with mCherry-*CSDE1* and EGFP-*MKRN2*, cultured 48 h. Images were acquired using the Olympus spinSR confocal microscope. Scale bar: 10 m. **(F–H)** Quantification of MKRN2 condensate number per cell, **(F)** verexpression of CSDE1 enhances SG formation, **(G)** nock out of *CSDE1* inhibits SG formation, **(H)** upplementation of CSDE1 partially rescued the phenotype caused by *CSDE1* deficiency, show as means ± SEM, *p < 0.05, **p < 0.01, ***p < 0.001, ****p < 0.0001. **(I)** FRAP analysis of CSDE1-mcherry condensates in HEK293 control or *MKRN2* KO cells. Images were acquired using the Olympus spinSR confocal microscope. Scale bar: 10 μm. **(J–L)** Quantification of FRAP in the bleached region of mCherry-CSDE1 condensates in HEK293 control or *MKRN2* KO cells, **(J)** *MKRN2* deficiency enhanced the fluorescence recovery of CSDE1(Ctrl *n* = 15; KO *n* = 17), **(K,L)** upplementation of MKRN2 partially rescued the phenotype caused by *MKRN2* deficiency (Ctrl *n* = 19; KO *n* = 17), show as means ± SEM, *p < 0.05, ****p < 0.0001. **(M)** LLPS of purified recombinant CSDE1 and MKRN2 in a gradient increasing concentration manner. After 5 min of PEG induction, images were acquired using the Zeiss LSM900 confocal microscope. Scale bar: 200 μμm. **(N,O)** Quantification of CSDE1 **(N)** or MKRN2 **(O)** condensate number for data in **(M)**. **(P)** Quantification of CSDE1 and MKRN2 condensate number for data in **(M)**.

We first generated EGFP–MKRN2 and mCherry-CSDE1 reporters (Supplementary Figure 2A). Under basal culture conditions only rare cells displayed micron-scale condensates (data not shown). We therefore challenged HEK293 or SH-SY5Y cells with 0.5 mM sodium arsenite to provoke oxidative stress. Within 30–60 min both proteins nucleated abundant cytoplasmic droplets that extensively overlapped (Figures 2A,E; Supplementary Figure 2D). Quantification revealed that co-expression of MKRN2 reduced the number of CSDE1-positive condensates per cell (Figure 2B), whereas CSDE1 co-expression increased MKRN2 droplet number (Figure 2F), implying that MKRN2 restricts CSDE1-driven SG formation while CSDE1 reciprocally stabilizes MKRN2 condensates.

To verify that these structures are bona fide LLPS assemblies we co-transfected GFP–G3BP1, the archetypal SG nucleator (Yang et al., 2020). G3BP1 colocalized almost completely with CSDE1 puncta (Supplementary Figure 2F). Time-lapse imaging showed fusion events and relaxation into rounded droplets, confirming liquid-like character. Droplet size and number varied markedly between cells, underscoring cellular heterogeneity in LLPS capacity.

We next exploited CRISPR KO lines to test genetic dependencies. Loss of *MKRN2* elevated CSDE1 condensate density; re-expression of wild-type MKRN2, but not its RING-deletion mutant, restored control levels (Figures 2C,D; Supplementary Figure 2E). Conversely, *CSDE1* deletion diminished MKRN2 droplet formation, and rescue with wild-type CSDE1, but not with the ubiquitination-defective 4KR mutant (K81/91/208/727R), reversed the defect (Figures 2G,H). Thus, the MKRN2–CSDE1 interaction and CSDE1 ubiquitination are required for their reciprocal regulation during LLPS.

Because CSDE1 recruits G3BP1 to nascent SGs (Guo et al., 2019), we enumerated G3BP1 droplets in KO cells. *CSDE1* absence increased G3BP1 puncta number, whereas *MKRN2* loss had no significant effect (Supplementary Figures 2G,H), indicating that CSDE1 directly modulates G3BP1 partitioning into condensates.

To probe droplet dynamics we performed FRAP on mCherry–CSDE1 puncta in HEK293 cells. Bleached regions recovered ~70% fluorescence within 90 s (Figures 2I–L), validating liquid-like properties. Recovery half-time was significantly shorter in *MKRN2*-KO cells (t½ ≈ 25 s) than in controls (t½ ≈ 45 s), and re-expression of MKRN2 lengthened t½ toward baseline (Figures 2K,L). Thus, MKRN2 attenuates CSDE1 molecular mobility within condensates, most likely by ubiquitin-dependent cross-linking or surface charge modulation.

Collectively, these data establish that MKRN2 and CSDE1 undergo oxidative-stress-induced LLPS, reciprocally regulate each other’s droplet formation, and that MKRN2-mediated ubiquitination fine-tunes condensate dynamics-potentially influencing the spatial translational control of CSDE1-bound mRNAs.

### 3.4 *In vitro* LLPS of CSDE1 and MKRN2

We next asked whether CSDE1 and MKRN2 can phase-separate in a minimal cell-free system. Prokaryotic expression vectors encoding mCherry*–CSDE1* and EGFP*–MKRN2* were constructed (Supplementary Figure 2I) and the fluorescent proteins were purified to homogeneity. Both proteins spontaneously formed spherical, liquid-like droplets under crowding conditions. After systematically varying buffer composition and PEG-20000 concentration, we selected 2% PEG as the standard crowding agent (Supplementary Figures 2J,K).

Titration of protein concentration revealed a steep, positive relationship between input concentration and droplet number for each protein alone (Figures 2M–O). Raising KCl from 50 to 600 mM produced a mild, non-significant increase in droplet count (Supplementary Figures 2L,M), indicating that electrostatic interactions contribute weakly to condensation under these conditions.

Strikingly, when both proteins were present simultaneously, the droplet formation of both CSDE1 and MKRN2 was strongly suppressed (≈ 60% reduction) (Figure 2P). Importantly, the two proteins never co-localized into the same droplets *in vitro*; instead, MKRN2 remained diffuse or formed separate, smaller foci that excluded CSDE1. Thus, in the minimal system, MKRN2 acts as a potent, dose-dependent antagonist of CSDE1 condensation rather than a co-partitioner.

The discrepancy with the co-condensation observed in cells implies that additional cellular factors—RNA, post-translational modifications, or auxiliary proteins (Ju Lee et al., 2017; Panas et al., 2016)—are required to license mixed CSDE1–MKRN2 droplets. Collectively, the cell-free data establish that MKRN2 can autonomously suppress CSDE1 phase separation through direct binding and/or steric interference, providing a biochemical basis for the ubiquitin-independent arm of the regulatory circuitry described *in vivo*.

### 3.5 Loss of function affects both MKRN2 and CSDE1 by reducing the amount of SG formation

We have confirmed that mutating the ubiquitination sites of CSDE1 reduces MKRN2 ubiquitination (Figures 1F,G). Truncated variants of the two proteins also display distinct interaction profiles (Figures 1H,I).

**Figure 3:**
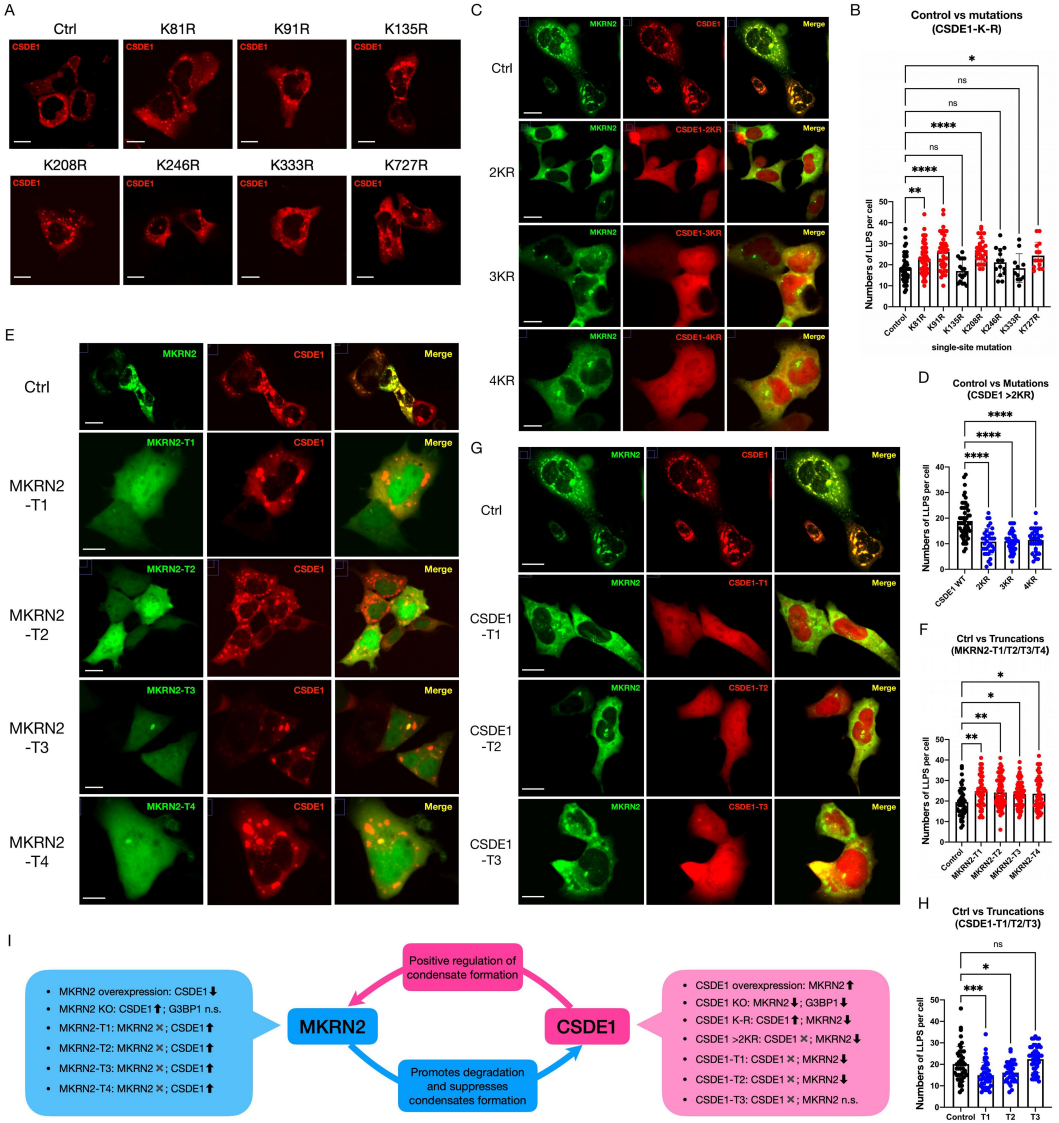
Loss of function affects both MKRN2 and CSDE1 by reducing the amount of SG formation. **(A)** Representative images of HEK293 cells expressing mCherry-CSDE1 with single mutations at seven different ubiquitination sites. Scale bar: 10 μm. **(B)** Quantification of CSDE1 condensate number per cell, single mutations at four major ubiquitination sites (K81, K91, K208, K727) significantly increased the condensate number. Show as means ± SEM, *p < 0.05, **p < 0.01, ****p < 0.0001. **(C)** Representative images of HEK293 cells expressing mCherry-CSDE1 with double (K81R/K91R), triple (K81R/K91R/K208R), and quadruple (K81R/K91R/K208R/K727R) mutations at ubiquitination sites. Scale bar: 10 μm. **(D)** Quantification of MKRN2 condensate number per cell, multi-site CSDE1 mutants significantly reduced MKRN2 condensate number. How as means ± SEM, ****p < 0.0001. **(E)** Representative images of HEK293 cells co-expressing EGFP-MKRN2 and truncated EGFP-MKRN2-T1/T2/T3/T4 with CSDE1. Scale bar: 10 m. **(F)** Quantification of CSDE1 condensate number per cell, co-expression of truncated MKRN2 with wild-type CSDE1 significantly increased the number of CSDE1 condensates. How as means ± SEM, *p < 0.05, **p < 0.01. **(G)** Representative images of HEK293 cells co-expressing mCherry-CSDE1 and truncated mCherry-CSDE1-T1/T2/T3 with MKRN2. Scale bar: 10 m. **(H)** Quantification of MKRN2 condensate number per cell, co-expression of truncated CSDE1 with wild-type MKRN2 significantly reduced the number of MKRN2 condensates. How as means ± SEM, *p < 0.05, ***p < 0.001, ns, not significant. **(I)** Schematic diagram of the interdependent regulation and negative feedback loop between MKRN2 and CSDE1 protein stability and condensate formation.

To dissect how these modifications affect phase separation, we generated fluorescently tagged plasmids bearing site-specific mutations or truncations (Supplementary Figure 3A,B). Single lysine-to-arginine substitutions at any of the seven ubiquitination sites did not block CSDE1 condensation (Figure 3A), yet mutations at four major sites (K81, K91, K208, K727) doubled condensate number in HEK293 cells (Figure 3B), identifying them as the principal MKRN2 targets. These single mutations impair MKRN2 ubiquitination activity without abolishing condensation, phenocopying *MKRN2* knockout (Figure 2D).

In stark contrast, combined mutations (double K81R/K91R, triple K81R/K91R/K208R, or quadruple K81R/K91R/K208R/K727R) completely eliminated CSDE1 phase separation. The protein lost cytoplasmic confinement and became dispersed throughout the cytoplasm and nucleus (Figure 3C), a mislocalization not seen in *CSDE1*-null cells. We infer that compromised ubiquitination perturbs CSDE1 structure—possibly exposing cryptic NLS/NES motifs (Köhler and Hurt, 2007) or weakening RNA binding (Guo et al., 2020)—thereby abrogating both localization and condensation. Co-expression of these multi-site mutants also reduced MKRN2 condensates (Figure 3D), mirroring the consequences of *CSDE1* depletion and confirming that proper ubiquitination is indispensable for CSDE1 structural integrity and phase-separation competence.

Truncations of either protein produced similar mislocalization: cytoplasmic fragments became diffuse and nuclear signal appeared (Figures 3E,G). These fragments formed ≤2 condensates per cell, some nuclear. Truncated MKRN2 elevated wild-type CSDE1 condensate number (Figure 3F), recapitulating *MKRN2* knockout (Figure 2D). Even the isolated C3H zinc-finger domain, though sufficient for CSDE1 binding (Figure 1J), was mislocalized and failed to condense, indicating that any structural deletion disrupts MKRN2 function. Conversely, truncated CSDE1 suppressed wild-type MKRN2 condensation (Figure 3H), resembling CSDE1 knockout (Figure 2H). The CSDE1-T3 fragment, which lacks both the cold-shock domain and ubiquitination sites, neither altered condensate number nor interacted detectably with MKRN2 (Figure 1I), underscoring that the CSD and intact ubiquitination sites are required for productive phase separation.

Collectively, our data establish a reciprocal regulatory circuit: MKRN2 ubiquitinates and destabilizes CSDE1 to limit condensate formation (RING-domain dependent), whereas CSDE1 promotes MKRN2 condensation. This creates a negative-feedback loop in which MKRN2 restrains its own positive regulator (Figure 3I). The structural integrity and ubiquitination status of both proteins are thus interlocked determinants of their phase behavior and subcellular distribution.

### 3.6 Loss of MKRN2 leads to abnormal social behaviors

Having established that MKRN2 and CSDE1 form an LLPS-dependent regulatory axis in both cellular and cell-free systems, and given that (i) CSDE1 is an established autism-spectrum-disorder (ASD) risk gene implicated in neuronal migration and differentiation (Guo et al., 2019; Kobayashi et al., 2013; Xia et al., 2014), (ii) germline *Csde1* deletion causes cortical malformation and embryonic lethality in mice (Jia et al., 2025), (iii) *MKRN2* inhibits neurogenesis in Xenopus (Yang et al., 2008), and (iv) dysregulated LLPS is increasingly linked to neurodevelopmental disorders (Shih et al., 2022; Mu et al., 2024; Zbinden et al., 2020; Ambadipudi et al., 2017; Ray et al., 2020; Ambadipudi et al., 2017), we asked whether *Mkrn2* loss-of-function in mice would elicit behavioral phenotypes relevant to neuropsychiatry.

**Figure 4:**
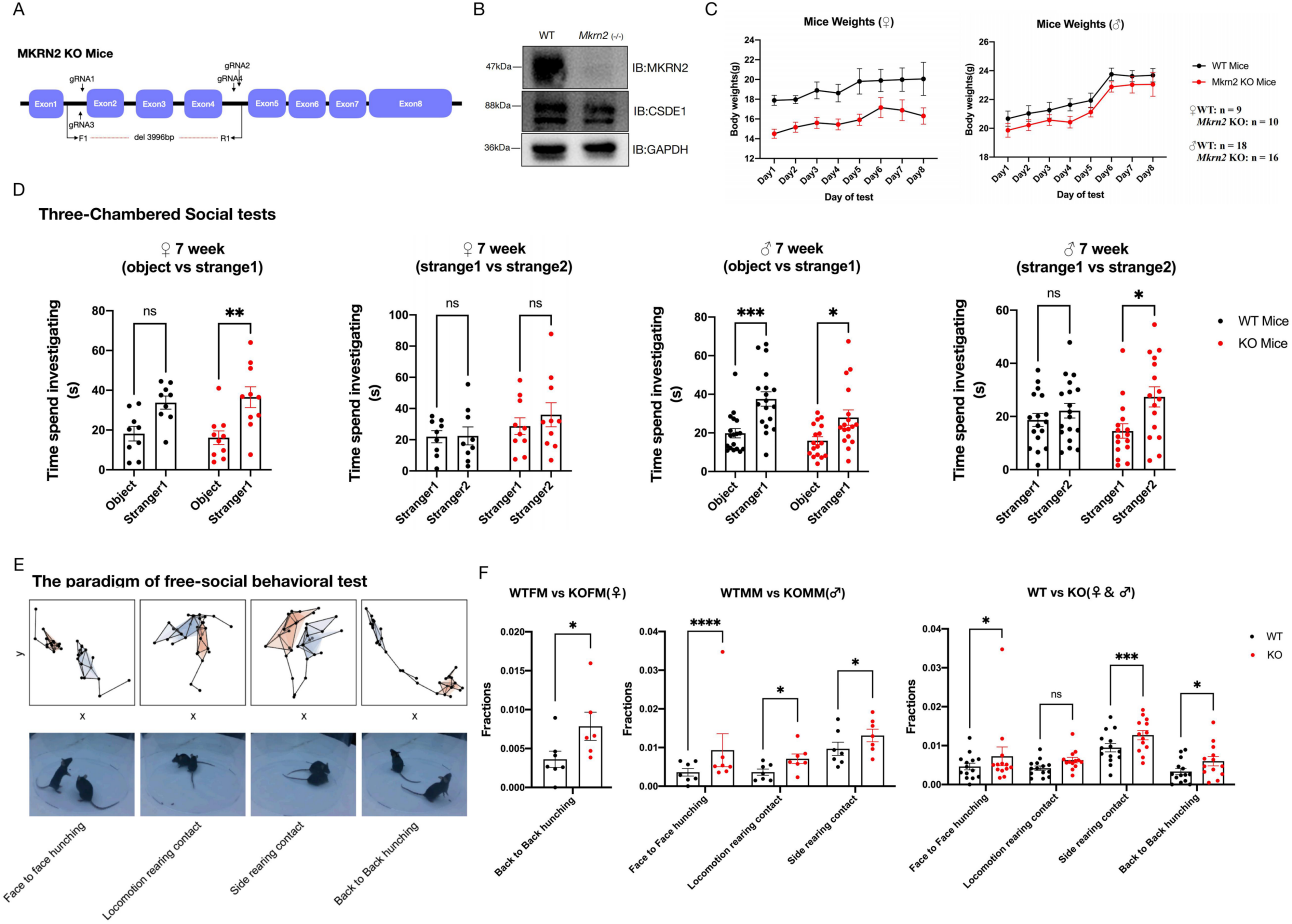
*Mkrn2* KO mice revealed sex-specific social abnormalities. **(A)** Schematic diagram of *Mkrn2* knockout mouse. **(B)** Representative western blots of MKRN2 proteins in the prefrontal cortex from 8 weeks mice. **(C)** Body weights of mice recorded everyday before behavior tests. **(D)** Time spent in each chamber for the sociability test performed with 2 groups of mice: ♀WT (*n* = 9), ♀*Mkrn2* KO (*n* = 10), ♂WT (*n* = 18), ♂*Mkrn2* KO (*n* = 16); show as means ± SEM, *p < 0.05, **p < 0.01, ***p < 0.001, n.s.: no significant difference. **(E)** Video acquisition for free social behavior test. The comparison of behavioral fractons of social behavioral modules of four social groups (♀/♂WT and ♀/♂ Mkrn2 KO mice freely social with respective stranger mice). The fractions of each group are normalized, and they are clustered and resorted according to the dimension of social behavior modules a total of 337 social behavior modules are identified. Social behavior modules with significant differences are manually identified. Orange 3D mice represent KO/WT testing mice, and gray 3D mice represent ool WT mice. **(F)** Quantification of 4 social behavior modules with significant differences are manually identified. KOFM/WTFM represent emale KO or WT mice, and KOMM/WTMM represent ale KO or WT mice show as means ± SEM, *p < 0.05, ***p < 0.001, ****p < 0.0001.

We acquired constitutive *Mkrn2*-knockout (KO) mice from Cyagen Biosciences and verified complete *Mkrn2* deletion by PCR genotyping and by loss of MKRN2 protein in prefrontal-cortex lysates (Figures 4A,B). Body weight was recorded before every behavioral session to control for non-specific effects (Figure 4C). Animals were then subjected to four validated paradigms: (1) self-grooming for repetitive behavior (Supplementary Figure 4A), (2) open-field for locomotion and anxiety-like behavior (Supplementary Figures 4C–E), (3) three-chamber social interaction for sociability and social novelty preference (Figure 4D), and (4) rotarod for motor coordination (Supplementary Figure 4B). No genotype differences emerged in grooming duration, center time, total distance, or rotarod latency, indicating that MKRN2 deletion does not perturb baseline locomotion, anxiety, repetitive behavior, or motor learning.

Strikingly, however, *Mkrn2*-KO mice displayed markedly enhanced sociability. During phase I of the three-chamber assay both genotypes preferred the stranger mouse over the empty cup, but KO animals exhibited significantly greater interaction time. In phase II, KOs showed a robust preference for the novel stranger (stranger 2) versus the now-familiar stranger 1, whereas controls did not, revealing exaggerated social novelty seeking (Figure 4D). Qualitatively similar trends were present in females but did not reach statistical significance.

To dissect social behavior with higher granularity, we partnered with the Hu Ji Laboratory (ShanghaiTech University) and the Wei Pengfei Laboratory (Shenzhen Institute of Advanced Technology) to perform 3D video-tracking of unrestrained social encounters (Supplementary Figure 4F; Han et al., 2023; Huang et al., 2021). Sex-specific divergence was immediately apparent (Figures 4E,F; Supplementary Figure 4G). Female KOs displayed increased back-to-back rearing and enlarged inter-individual distance during rearing bouts-ethological signatures of social withdrawal that parallel the passive avoidance phenotype often observed in autistic girls (Dean et al., 2017; Mandy and Tchanturia, 2015). Conversely, male KOs exhibited excessive face-to-face hunching and elevated rear contact while in motion, reflecting abnormally intrusive approach behavior reminiscent of the “forced social proximity” and poor personal-space regulation seen in a subset of autistic boys (May et al., 2014).

Collectively, our data demonstrate that *Mkrn2* deficiency in mice produces nuanced, sex-dimorphic alterations in social repertoire that mirror the qualitative heterogeneity of human ASD. The specificity of the phenotype-sparing motor, anxiety, and repetitive domains-positions *Mkrn2* loss as a selective driver of social-circuit dysfunction rather than a global neurodevelopmental insult. The recapitulation of male-biased penetrance and female-specific expressivity further validates the model for mechanistic dissection of sex-specific pathways underlying ASD.

### 3.7 Identification of ASD-relevant mRNA candidates regulated by the MKRN2-CSDE1 axis

The observed social deficits in *Mkrn2*-KO mice prompted us to investigate the cellular mechanisms downstream of its substrate, CSDE1. Given CSDE1’s established role as an RNA-binding protein that drives stress-granule assembly (Youn et al., 2018) and selectively remodels the translatome (Chang et al., 2004; Guo et al., 2020; Kakumani et al., 2020), we asked whether its messenger-RNA partners also regulate, or are regulated by, CSDE1 phase separation. mRNA itself can act as a multivalent scaffold that accelerates RBP condensation, especially when translation stalls and unprocessed transcripts accumulate (Panas et al., 2016). We therefore set out to identify the CSDE1-bound transcriptome under conditions in which CSDE1 ubiquitination by MKRN2 is either intact or abolished.

We performed RIP-seq in *MKRN2*-knockout SH-SY5Y cells stably expressing Flag-tagged CSDE1. Three isogenic conditions were compared: (i) empty vector, (ii) CSDE1-Flag alone, and (iii) CSDE1-Flag plus MKRN2-Myc rescue. Anti-Flag immunoprecipitates were subjected to strand-specific library preparation and 2 × 150-bp paired-end sequencing (Genewiz). After stringent peak calling (CLIPper, FDR ≤ 0.01) and differential-binding analysis (DESeq2), ~ 20,000 transcripts showed CSDE1 occupancy that was reproducibly shifted ≥ 1.5-fold in the absence of *MKRN2*. Effect sizes were modest (|log₂FC| < 2.5 for 90% of targets), consistent with fine-tuning rather than on/off regulation.

**Figure 5:**
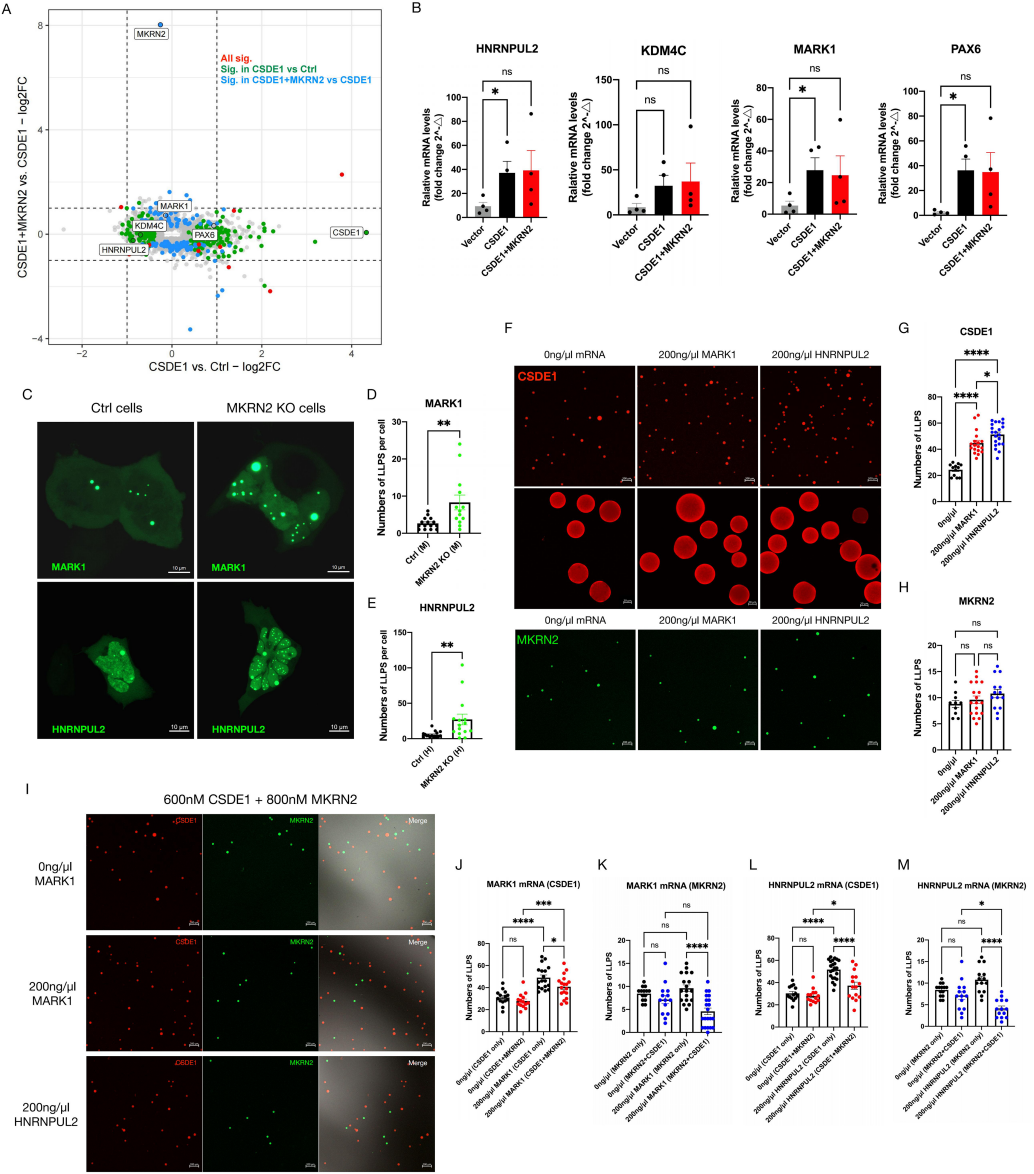
ASD-relevant mRNAs induce LLPS of CSDE1 and MKRN2 *in vitro*. **(A)** Cross-referenced the RIP sequencing data with the SFARI autism gene database. Screen out four high-confidence ASD-associated genes exhibiting CSDE1 ubiquitination-dependent regulation: *MARK1, PAX6, KDM4C*, and *HNRNPUL2*. **(B)** Analysis with RT-qPCR for the expression level of selected mRNAs in MKRN2 KO SH-SY5Y cells. **(C)** Expression of *MARK1* and *HNRNPUL2* mRNAs in HEK293 control and *MKRN2* KO cells using MS2-MCP RNA reporter system. Images were acquired using the Zeiss LSM900 confocal microscope after 48 h of transfection. Scale bar: 10 μm. **(D,E)** Quantification of *MARK1* and *HNRNPUL2* mRNAs condensate number for data in **(C)**. **(F)** LLPS of purified recombinant CSDE1 (600 nM) or MKRN2 (800 nM) with 200 ng/μL *MARK1* or *HNRNPUL2* mRNAs. After 5 min of induction, images were acquired using the Zeiss LSM900 confocal microscope. Scale bar: 200 and 20 μm. **(G,H)** Quantification of CSDE1 **(G)** or MKRN2 **(H)** condensate number for data in **(F)**. **(I)** LLPS of purified recombinant CSDE1 and MKRN2 with 200 ng/μL *MARK1* or *HNRNPUL2* mRNAs. After 5 min of induction, images were acquired using the Zeiss LSM900 confocal microscope. Scale bar: 200 μm. **(J,K)** Quantification of CSDE1 **(J)** and MKRN2 **(K)** condensate number after 200 ng/μL *MARK1* mRNA induction for data in **(I)**. **(L,M)** Quantification of CSDE1 **(L)** and MKRN2 **(M)** condensate number after 200 ng/μL *HNRNPUL2* mRNA induction for data in **(I)**. All quantification data show as means ± SEM, *p < 0.05, **p < 0.01, ***p < 0.001, ****p < 0.0001, ns, not significant.

Cross-referencing these data with the SFARI autism gene database yielded 117 overlapped ASD genes (Supplementary Figure 5A). Recognizing that RIP-seq captures the CSDE1-containing complex (which may include both direct and MKRN2-mediated RNA associations), we integrated this list with neurobiological literature to prioritize candidates with high relevance. After applying filters (mean TPM ≥ 10, FDR ≤ 0.05, proteomic support), *HNRNPUL2* and *KDM4C* were identified (Figure 5A). To broadly probe MKRN2–CSDE1 roles in ASD pathways, we expanded the list to 10 additional neurodevelopmentally relevant genes, including *MARK1* (synaptogenesis) (Maussion et al., 2008; Kelly-Castro et al., 2024) and *PAX6* (neurogenesis) (Kikkawa et al., 2019; Divya et al., 2016; Kawada et al., 2018), despite their lower statistical significance in the RIP-seq data (Figure 5A; Supplementary Figure 5A).

Targeted qPCR validation of these 12 candidates confirmed reproducible enrichment of *HNRNPUL2*, *MARK1*, and *PAX6* in CSDE1 immunoprecipitates (Figure 5B; Supplementary Figure 5B). Notably, the direction of change in these focused assays did not always align with the initial genome-wide trends, highlighting that our RIP-seq data identify mRNAs whose association with the CSDE1 complex is subject to MKRN2-dependent regulation, rather than predicting the exact outcome of all downstream validations. Given their established neurobiological relevance—*HNRNPUL2* as a high-confidence ASD gene and *MARK1* as a key synaptogenesis regulator—and their reproducible association with CSDE1, we selected *HNRNPUL2* and *MARK1* for in-depth mechanistic investigation.

To visualize the transcripts in living cells, we inserted 24 × MS2 stem–loops into the 3′ UTR of each gene and co-expressed MCP-GFP together in control or *MKRN2*-knockout HEK293 cells. Live-cell imaging revealed that all four mRNAs formed discrete, spherical nuclear foci (Figure 5C; Supplementary Figure 5C). Quantitative single-molecule RNA-FISH showed a 1.7- and 2.1-fold increase in *MARK1* and *HNRNPUL2* focus number per nucleus in *MKRN2*-KO cells (*p* < 0.01) (Figures 5D,E), whereas *KDM4C* and *PAX6* trends did not reach significance (Supplementary Figure 5D). Under these experimental conditions, CSDE1 was predominantly cytoplasmic (as shown in Figures 2A,E; Supplementary Figure 2D), indicating a spatial separation from the nuclear mRNA foci. These data demonstrate that MKRN2 deficiency specifically alters the subnuclear condensation state or abundance of HNRNPUL2 and MARK1 mRNAs.

### 3.8 Candidate mRNAs modulate the condensation dynamics of the MKRN2-CSDE1 complex

We next asked whether these specific mRNAs could directly modulate the core biophysical property of the MKRN2-CSDE1 axis: biomolecular condensation. To address this, we reconstituted phase separation *in vitro* with purified components. Full-length *MARK1* and *HNRNPUL2* mRNAs were synthesized by T7 runoff transcription, capped, poly-adenylated, and gel-purified before use.

Titration of *MARK1* mRNA (0–500 ng μL^−1^) into 0.6 μM CSDE1 at 150 mM KCl revealed a biphasic response: condensate number rose steeply between 50 and 200 ng μL^−1^, plateaued, and declined at ≥500 ng μL^−1^, consistent with RNA-driven cross-linking followed by solubilization at excess RNA (Supplementary Figures 5E,F). *MKRN2* droplet counts remained unchanged across the same interval (Supplementary Figure 5G), indicating that *MARK1* mRNA does not act as a general phase-separation scaffold. All subsequent assays were performed at 200 ng μL^−1^ RNA, the lowest concentration that saturated CSDE1 droplet yield.

Both *MARK1* and *HNRNPUL2* mRNAs specifically lowered the critical concentration of CSDE1 and increased droplet number at fixed protein concentration (Figures 5F,G). *HNRNPUL2* mRNA was consistently more potent than *MARK1* mRNA (1.6-fold vs. 1.3-fold increase; *p* = 0.045, one-way ANOVA). Neither transcript altered MKRN2 droplet formation (Figure 5H), confirming selectivity.

We then tested whether MKRN2 could antagonize this RNA-driven CSDE1 condensation (Figure 5I). In the absence of RNA, co-incubating CSDE1 and MKRN2 had no significant effect. Addition of *MARK1* mRNA triggered robust CSDE1 droplets that were reduced by 42 ± 5% when MKRN2 was present (Figure 5J). MKRN2 droplet counts stayed constant (Figure 5K), suggesting ubiquitination of CSDE1—rather than direct RNA binding—dampens condensation. An identical experiment with *HNRNPUL2* mRNA produced an even stronger suppression (58 ± 4% reduction) (Figure 5L), paralleling the higher basal potency of this transcript. Concomitantly, MKRN2 droplets were diminished only when CSDE1 was co-expressed (Figure 5M), indicating reciprocal exclusion from the RNA-rich phase.

Together, this in vitro reconstitution shows that (i) *MARK1* and *HNRNPUL2* mRNAs act as selective scaffolds for CSDE1 condensates, and (ii) MKRN2-mediated ubiquitination reverses RNA-driven phase separation, providing a direct mechanistic link between post-translational modification and condensate dynamics regulated by ASD-relevant mRNAs.

## 4 Discussion

Our study elucidates a novel liquid–liquid phase separation (LLPS)-dependent regulatory mechanism in which MKRN2-mediated ubiquitination controls CSDE1 function, with implications for autism spectrum disorder (ASD). We discuss these findings in the context of phase separation biology, E3 ligase function, and neurodevelopmental disorders.

### 4.1 A reciprocal, LLPS-dependent regulatory relationship

MKRN2 inhibits CSDE1 condensation and reduces its dynamics, whereas CSDE1 promotes MKRN2 droplet formation, suggesting co-regulation for maintaining stress granule homeostasis (Youn et al., 2018; Guo et al., 2019). The discrepancy between cellular co-condensation and *in vitro* suppression indicates that their interaction dynamically modulates the energy landscape of phase separation, potentially requiring cellular cofactors such as RNA (Ju Lee et al., 2017; Panas et al., 2016). This aligns with emerging roles of E3 ligases in regulating condensate dynamics via ubiquitination (Yasuda et al., 2020; Li et al., 2023). We propose that MKRN2 may sterically inhibit CSDE1 self-assembly through ubiquitination or form an alternative soluble complex. The RNA-rich cellular environment likely overcomes this suppression, enabling coordinated phase separation and highlighting the critical influence of biological context on phase separation regulation (Langdon et al., 2018).

### 4.2 Ubiquitination dictates CSDE1 localization and function

The mislocalization of CSDE1 ubiquitination-site mutants underscores that ubiquitination is crucial for its proper cytoplasmic retention. Loss of ubiquitination may induce conformational changes, disrupt nuclear export, or interfere with cytoplasmic anchoring, leading to aberrant nuclear diffusion (Köhler and Hurt, 2007). This mislocalization directly compromises CSDE1’s phase separation capability, highlighting a primary mechanism by which MKRN2 regulates its function. While our work definitively establishes MKRN2 as the E3 ligase for CSDE1, the precise functional consequence—whether regulating protein stability via the proteasome or altering its interactome or activity—remains an exciting avenue for future study.

### 4.3 A composite interaction interface

Our mapping reveals that the central region of MKRN2, encompassing multiple CCCH zinc finger motifs, is involved in CSDE1 binding. The T1 fragment (with three zinc fingers) is a major driver, while the T4 fragment (with one zinc finger) also showed binding capacity in initial assays (Laity et al., 2001). The inability to consistently validate binding with isolated T4 may reflect its loss of native conformation outside the full-protein context. This suggests that multiple motifs may cooperatively form a composite binding interface within the full-length protein, a hypothesis awaiting structural validation.

### 4.4 Targeting specific mRNAs within a dynamic complex

A key finding is the MKRN2-dependent regulation of specific ASD-relevant mRNAs, *HNRNPUL2* and *MARK1*. Our RIP-seq data, which profile the CSDE1-containing ribonucleoprotein complex, showed that MKRN2 loss modestly reshapes its RNA content. However, targeted qPCR validation revealed a pronounced increase in CSDE1 association with these specific candidates, a trend corroborated by increased nuclear mRNA foci in *MKRN2*-KO cells. This apparent discrepancy likely arises from methodological differences: RIP-seq provides a population-averaged, static snapshot of complex composition, which can be influenced by indirect transcriptional changes and buffering within the RNP network. In contrast, hypothesis-driven validation may more sensitively capture the functional regulation of specific high-affinity interactions. Therefore, we prioritized candidates by integrating the RIP-seq dataset (as an indicator of complex membership) with independent criteria of established neurobiological relevance (e.g., SFARI database, synaptic function), rather than relying solely on statistical significance from the sequencing data alone. Notably, these mRNAs form nuclear foci, while CSDE1 is cytoplasmic under steady-state conditions. This spatial separation, coupled with their robust biochemical association, suggests a dynamic or mediated interaction model rather than stable co-condensation. It posits that the MKRN2-CSDE1 complex may function as a regulatory hub, where MKRN2 could influence CSDE1’s nucleocytoplasmic shuttling or its partnership with other nuclear RNA-binding proteins to engage target mRNAs.

### 4.5 Link to *in vivo* phenotype and ASD pathobiology

The physiological relevance of this axis is underscored by sex-dimorphic social deficits in *Mkrn2*-KO mice, which resemble the heterogeneity of human ASD (Dean et al., 2017; Mandy and Tchanturia, 2015; May et al., 2014). Given CSDE1’s status as an ASD risk gene (Guo et al., 2019; Xia et al., 2014) and its regulation of ASD-associated mRNAs (e.g., *MARK1*, *HNRNPUL2*), dysregulation of the MKRN2-CSDE1 complex within neuronal condensates could disrupt synaptic plasticity and neural circuit function, core aspects of neurodevelopmental disorders (Guo et al., 2019; Zeng et al., 2016).

### 4.6 Limitations and future directions

We acknowledge certain limitations. The RIP-seq data reflect the RNA content of the CSDE1 complex but cannot resolve direct versus indirect associations. Furthermore, while our quantitative analysis focused on condensate number as a key metric, a comprehensive biophysical characterization—including size distribution, density, and internal architecture—remains for future investigation. Our *in vitro* LLPS assays, while providing controlled mechanistic insights, use simplified conditions that may not fully recapitulate the cellular milieu. Future efforts should therefore focus on: (1) validating the spatial and functional dynamics of this axis in primary neurons *in vivo*; (2) determining the precise consequence of CSDE1 ubiquitination (e.g., on protein stability via degradation assays); and (3) elucidating the structural basis of the MKRN2-CSDE1 interaction. Such studies will be crucial for exploring this pathway as a potential therapeutic target.

### 4.7 Translational outlook and deepened mechanistic inquiry

Looking forward, the MKRN2-CSDE1 axis presents rich opportunities for both mechanistic dissection and therapeutic exploration. To move beyond correlation toward causation, future studies could employ advanced in vivo imaging (e.g., two-photon microscopy) to directly visualize the dynamics of this complex in living neurons during synaptic activity. Reconstitution experiments incorporating neuronal lysates or specific RNA cohorts could identify the bona fide cofactors enabling physiological co-condensation. Furthermore, the sex-dimorphic behavioral phenotypes in *Mkrn2*-KO mice provide a unique entry point to dissect sex-specific molecular underpinnings of ASD-related social circuits. Ultimately, this pathway’s dependence on LLPS makes it uniquely tractable for targeted modulation. High-throughput screening for small molecules that correct pathological phase separation driven by MKRN2 deficiency could yield novel therapeutic leads for neurodevelopmental disorders.

In summary, we identify the MKRN2-CSDE1 axis as a novel LLPS-coupled ubiquitination pathway that fine-tunes the condensation state of a key RBP and its association with specific neuronal mRNAs. Dysregulation of this axis disrupts granular dynamics and mRNA metabolism, contributing to the complex social behavioral phenotypes observed in ASD models, thereby highlighting a new target for mechanistic and therapeutic exploration in neurodevelopmental disorders.

## 5 Conclusion

In conclusion, our study demonstrates that MKRN2 acts as a novel E3 ubiquitin ligase for the ASD risk factor CSDE1, mediating its ubiquitination at four conserved lysine residues in a liquid–liquid phase separation (LLPS)-dependent manner. Mkrn2 deficiency in mice leads to sex-specific social behavioral abnormalities reminiscent of ASD. Mechanistically, we establish that the MKRN2-CSDE1 axis regulates the condensation and interaction of specific neurodevelopmental mRNAs, including HNRNPUL2 and MARK1, and can antagonize mRNA-driven CSDE1 condensation in vitro. These findings converge into a model wherein dysregulation of this LLPS-coupled ubiquitination pathway disrupts precise RNA-protein interaction dynamics, potentially contributing to neurodevelopmental disorder pathogenesis. This work unveils a previously unrecognized regulatory node at the interface of post-translational modification, biomolecular condensation, and mRNA metabolism, offering new mechanistic insights for understanding neuropsychiatric disorders.

## Data Availability

The datasets generated and analyzed during the current study are available in the following public repositories: 1. The mass spectrometry proteomics data for ubiquitination sites on CSDE1 have been deposited to the ProteomeXchange Consortium via the PRIDE partner repository with the dataset identifier “PXD073844”. 2. The RIP-seq data have been deposited in the Gene Expression Omnibus (GEO) database under the accession number “GSE317793”. 3. All raw and analyzed data from the mouse 3D free social interaction experiments, including video recordings and summary files, have been deposited on Zenodo and are accessible via the persistent DOI: “10.5281/zenodo.18362509”.

## Footnotes

1
http://www.uniprot.org/
