# Supplementary material for: Liquid–liquid phase separation couples MKRN2-mediated ubiquitination of CSDE1 with neurodevelopmental disorders
Source: Front Cell Neurosci. 2026 Feb 11;20:1757304. doi: 10.3389/fncel.2026.1757304 (PMC12932526; doi:10.3389/fncel.2026.1757304)
Supplement: Supplementary file 1 [file Data_Sheet_1.ZIP › Supplementary_Material-Zi Wang /Supplementary_Material-Zi Wang.docx]

Supplementary Material

# Supplementary Figures


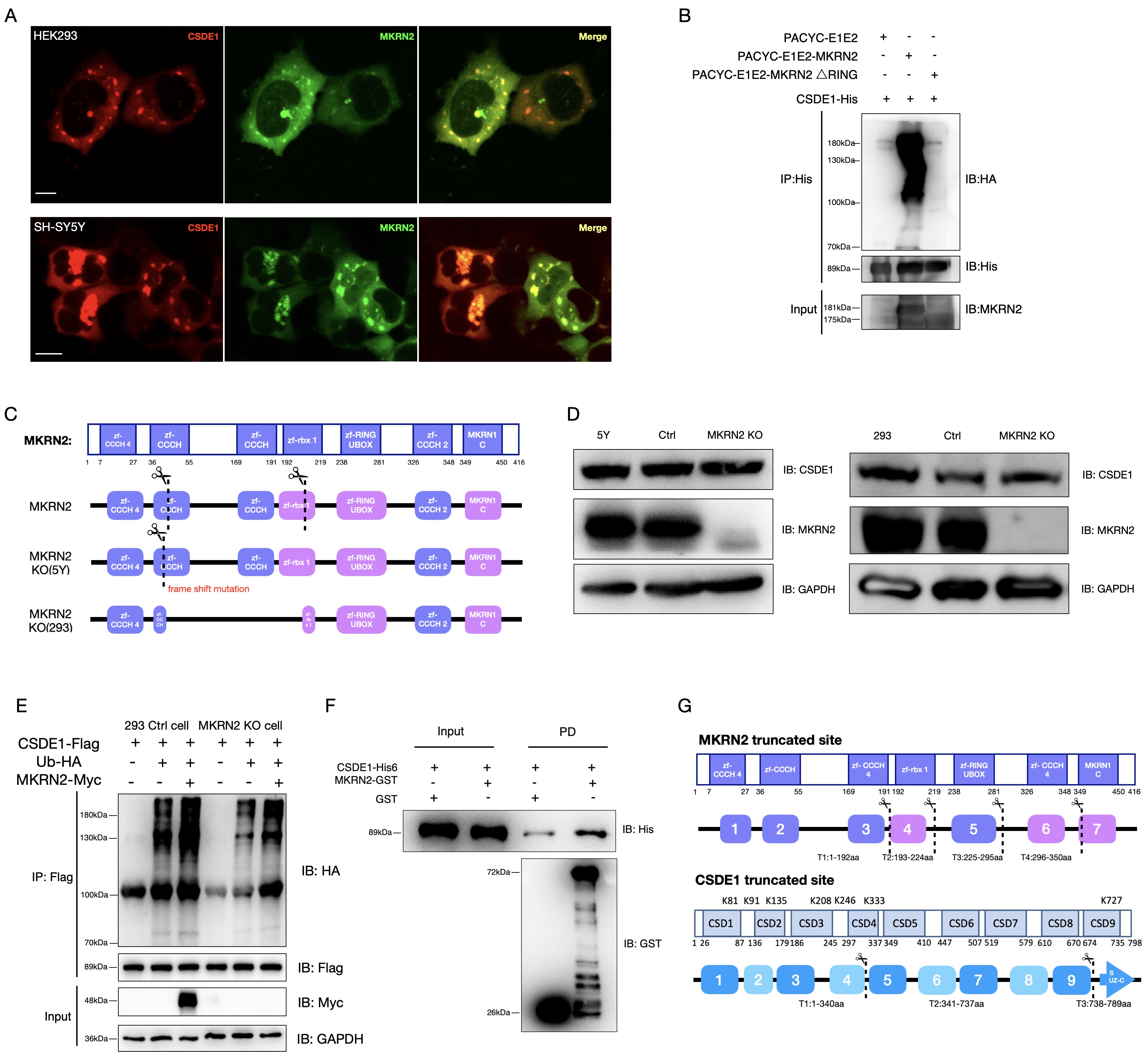


**Supplementary Figure 1.** Human MKRN2 interacts with CSDE1 in vitro and in vivo. **(A)** Co-localization of MKRN2 and CSDE1 in HEK293 and SHSY5Y cells. Both cells were transfected with EGFP-MKRN2 and mCherry-CSDE1, after 48h cells were exposed to sodium arsenite (500 mM; 30min) before observation. Images were acquired using the Olympus spinSR confocal microscope. Scale bar: 10 μm.**(B)** The RING domain of MKRN2 is essential for the ubiquitination of CSDE1. MKRN2 ubiquitinates CSDE1 in vitro, with CSDE1-His, PACYCE1-E1E2-MKRN2, or PACYCE1-E1E2-Mkrn2(△RING)- Myc; 48 h later, the cell lysates were immunoprecipitated with anti-Flag affinity gels and subjected to immunoblotting analysis. **(C)** Schematic diagram of WT MKRN2, 5Y MKRN2 KO (SHSY5Y: frame shift mutation), and 293 MKRN2 KO (HEK293: large knockout).**(D)** Western blot of MKRN2 and CSDE1 proteins in the SH-SY5Y and HEK293 MKRN2 knockout cell lines. **(E)** CSDE1 ubiquitination is reduced in HEK293 MKRN2 KO cells compared to wild-type cells. Reintroduced MKRN2 can rescue CSDE1 ubiquitination. MKRN2 KO or wild-type HEK293 cells were co-transfected with HA-Ub, Csde1-Flag and/or MKRN2-Myc as indicated.**(F)** The direct interaction of MKRN2 with CSDE1 was detected by the GST pull-down assay. Reconstituted GST-tagged MKRN2 and His-tagged CSDE1 were purified, and the GST pull-down assay was performed and detected by immunoblotting. PD, GST pull-down. **(G)** Schematic diagram of MKRN2 and CSDE1 with truncated sites.


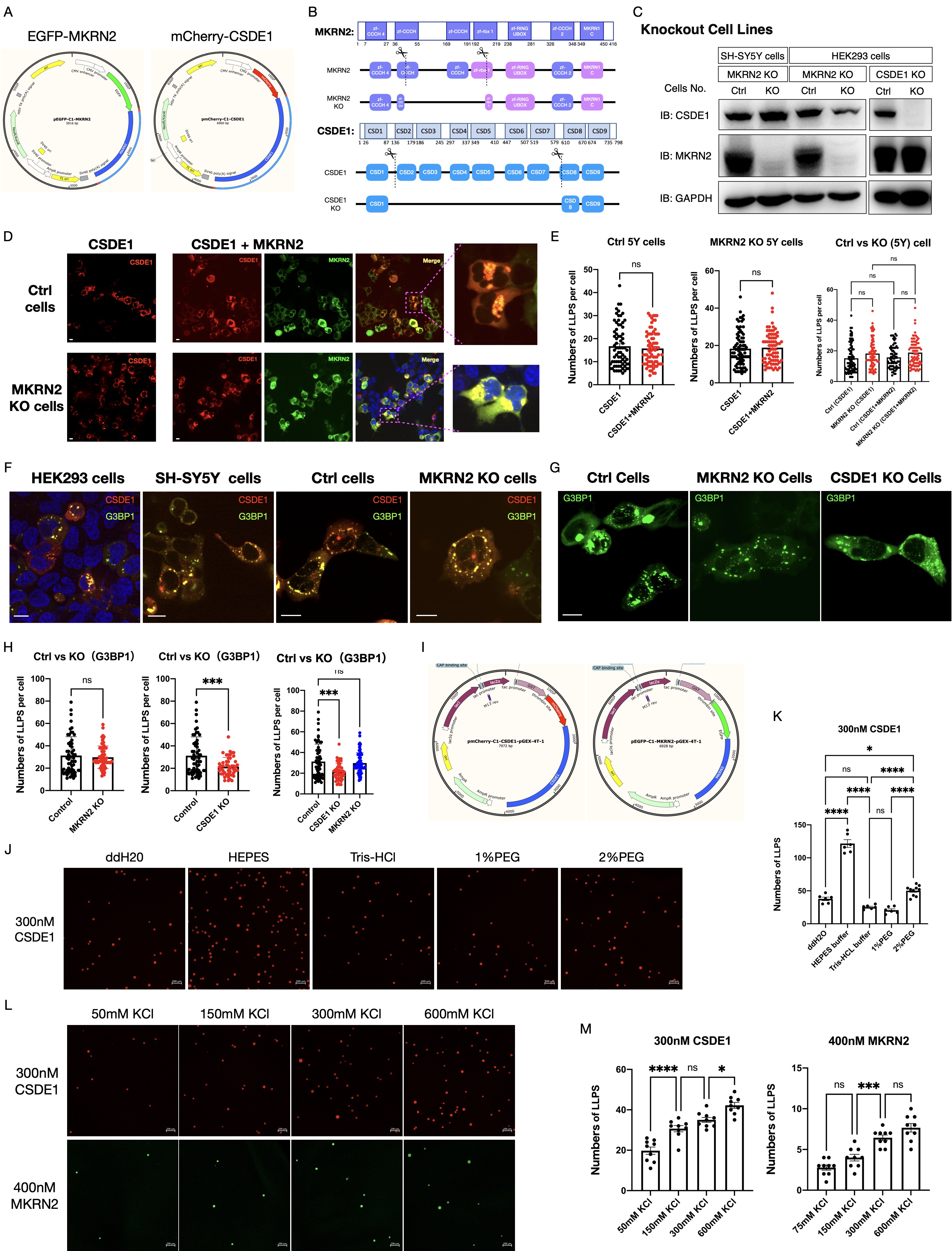


**Supplemental Figure 2.** Numbers of SG formation relies on MKRN2 and CSDE1 interactions in vivo & in vitro. **(A)** Schematic diagram of EGFP-MKRN2 and mCherry-CSDE1 constructs for in vivo experiments. **(B)** Schematic diagram of MKRN2 knockout, and CSDE1 knockout in HEK293 cells. **(C)** Western blot of MKRN2 and CSDE1 proteins in the MKR2 and CSDE1 knockout cell lines. **(D)** CSDE1 and MKRN2 are recruited to SGs under oxidative stress. SH-SY5Y WT and MKRN2 knockout cells treated with sodium arsenite(500μM, 30min) were overexpressed with mCherry-CSDE1(red) and EGFP-MRN2 (green). After 48h, images were acquired using the Olympus spinSR confocal microscope. Scale bar: 10 μm.**(E)** Quantification of CSDE1 condensate number per cell from(D),  overexpression of MKRN2 slightly inhibits SG formation. show as means ± SEM, ns: not significant. **(F)** CSDE1 and G3BP1 are recruited to SGs under oxidative stress. SH-SY5Y, HEK293 WT, HEK293 Control and MKRN2 knockout cells treated with sodium arsenite(500μM, 30min) were overexpressed with mCherry-CSDE1(red) and EGFP-G3BP1 (green). After 48h, images were acquired using the Olympus spinSR confocal microscope. Scale bar: 10 μm. **(G)** G3BP1 alone is induced under oxidative stress. HEK293 Control, MKRN2 knockout and CSDE1 knockout cells treated with sodium arsenite(500μM, 30min) were overexpressed with EGFP-G3BP1 (green). After 48h, images were acquired using the Olympus spinSR confocal microscope. Scale bar: 10 μm. **(H)** Quantification of G3BP1 condensate number per cell,  Knockout of CSDE1 inhibits G3BP1 SG formation. show as means ± SEM, ns: not significant, ***P < 0.001. **(I)** Schematic diagram of mCherry-CSDE1, and EGFP-MKRN2 constructs for in vitro experiments. **(J)** LLPS of purified recombinant CSDE1 in different buffers and concentrations of the crowding agent PEG20000. After 5min of induction, images were acquired using the Zeiss LSM900 confocal microscope. Scale bar: 200μm. **(K)** Quantification of CSDE1 condensate number for data in (J).  show as means ± SEM, *P < 0.05, ****P < 0.0001, ns: not significant.**(L)** LLPS of purified recombinant CSDE1 in 2%PEG buffer with different concentrations of KCl. After 5min of induction, images were acquired using the Zeiss LSM900 confocal microscope. Scale bar: 200μm.**(M)** Quantification of CSDE1 and MKRN2 condensate number for data in (L).  show as means ± SEM, datas not significant.


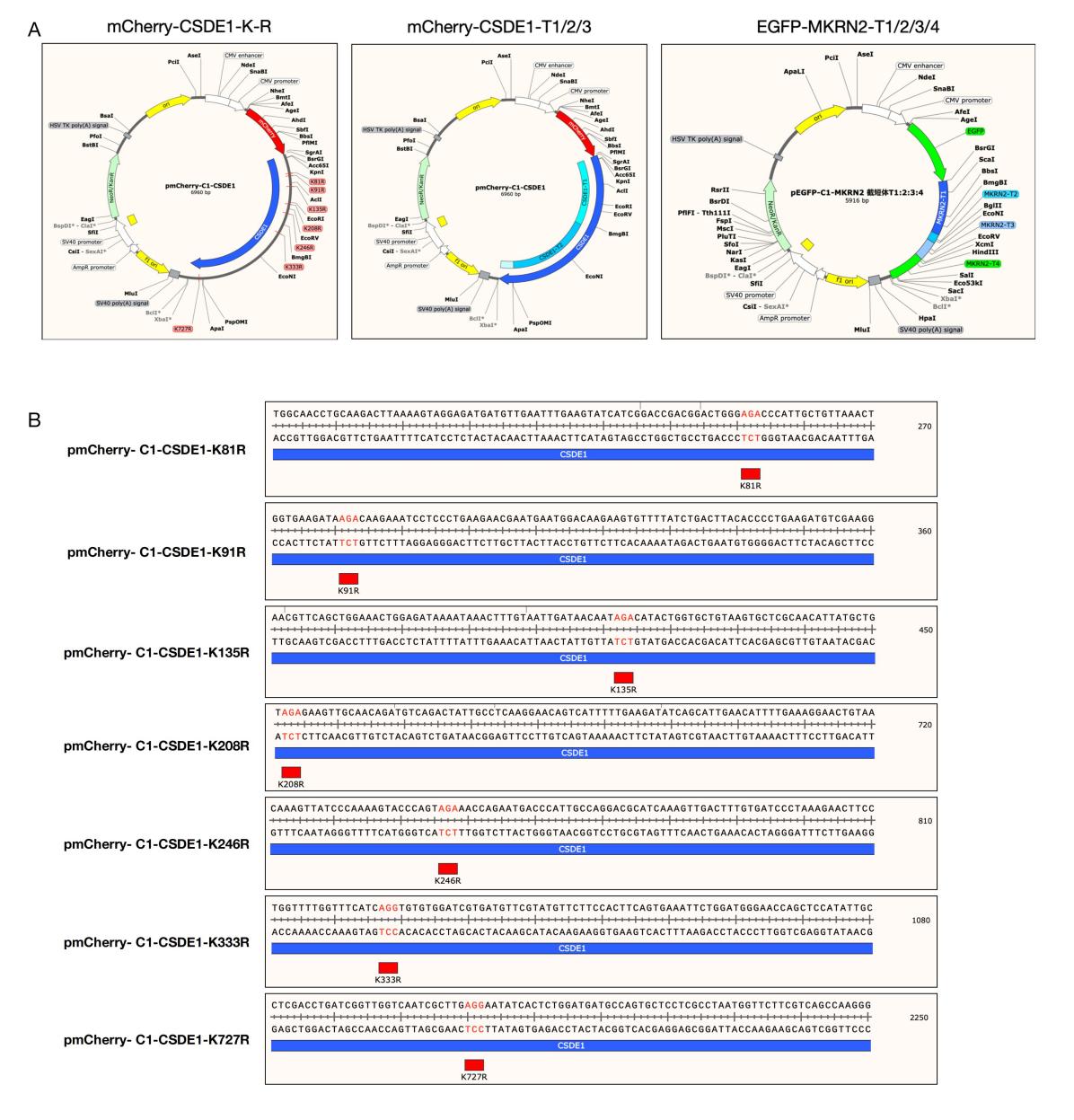


**Supplemental Figure 3.** Loss of function affects both MKRN2 and CSDE1 by reducing the amount of SG formation. **(A)** Schematic diagram of single site mutation of mCherry-*CSDE1*-K-R, truncated mCherry-*CSDE1*-T1/T2/T3 and truncated EGFP-*MKRN2*-T1/T2/T3/T4 constructs. **(B)** Sequencing check of constructed plasmids.


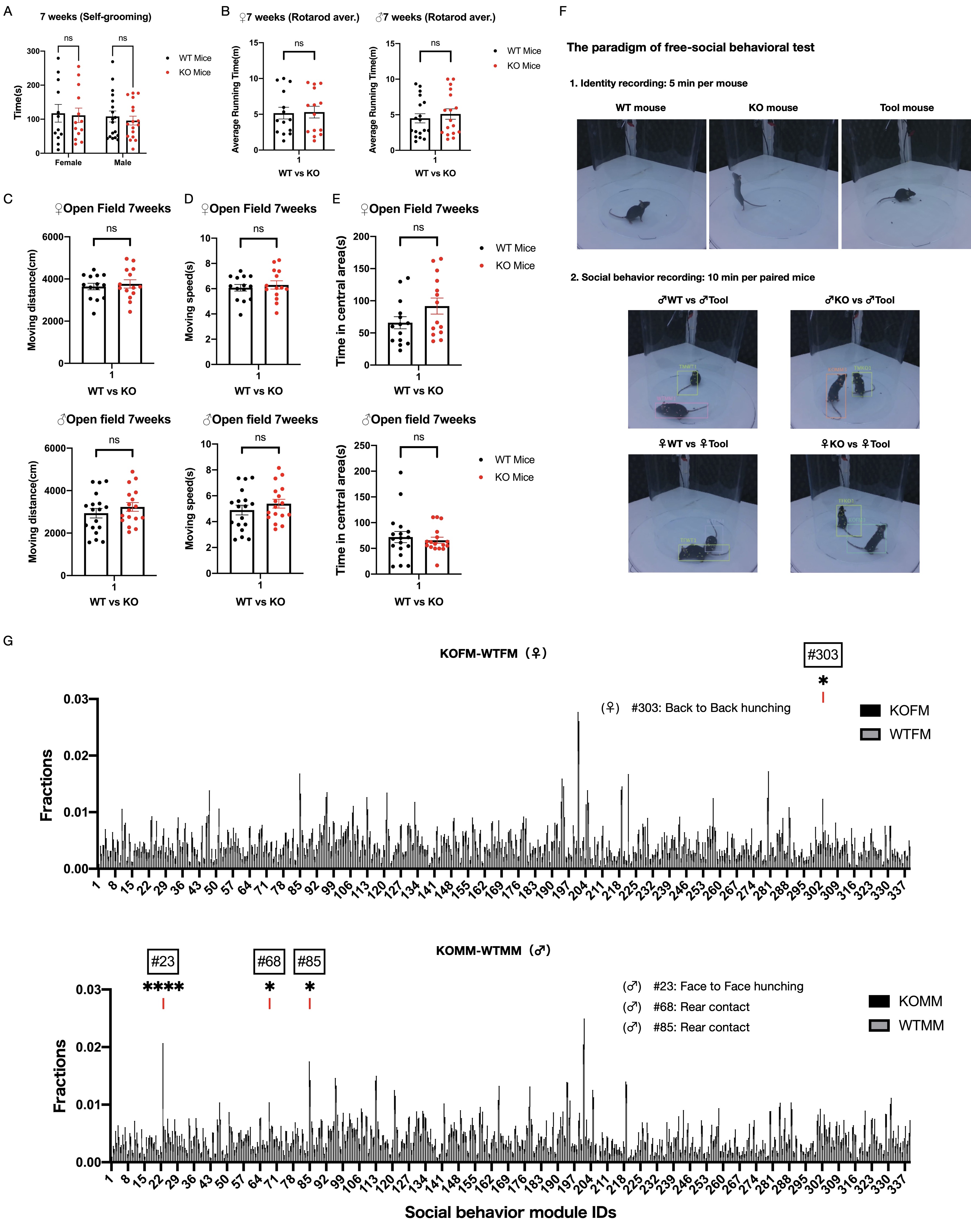


**Supplemental Figure 4.** Mkrn2 KO mice revealed sex-specific social abnormalities. **(A)** Time spent in self-grooming for repetitive behavioral test performed with 2 groups of mice. ♀WT(n = 9), ♀Mkrn2 KO (n = 10), ♂WT (n = 18), ♂Mkrn2 KO (n = 16). **(B)** Time of running in the rotarod test performed with 2 groups of mice. ♀WT(n = 14), ♀Mkrn2 KO (n = 14), ♂WT (n = 18), ♂Mkrn2 KO (n = 17). **(C)** Representative activity traces of total moving distance in the open-field test performed with 2 groups of mice. Same ‘n’ as in rotaod test. **(D)** Representative activity traces of average speed in the open-field test performed with 2 groups of mice. **(E)** Representative activity traces of time spent in center in the open-field test performed with 2 groups of mice.**(F)** Video acquisition for free social behavior test. Four cameras are located at four corners for behavioral capturing, and it is calibrated by checkboard images. There  are two phase for beahvioral video capturing including social behavior test and animal digital identity. The phase 1 is to capture identities of each mice. The phase 2 is to capture the videos of free-social interactions of two mice. **(G)** The comparison of  behavioral fractons  of social behavioral modules of four social groups (♀ / ♂WT and ♀ / ♂ Mkrn2 KO mice freely social with respective stranger mice). The fractions of each group are normalized, and  they are clustered and resorted according to the dimension of social behavior modules, A total of 337 social behavior modules are identified. 4 social behavior modules with significant differences are manually identified. KOFM / WTFM represent Female KO or WT mice, and KOMM / WTMM represent Male KO or WT mice. All data are presented as means ± SEM; *: P<0.05, ****: P<0.0001, ns: not significant.


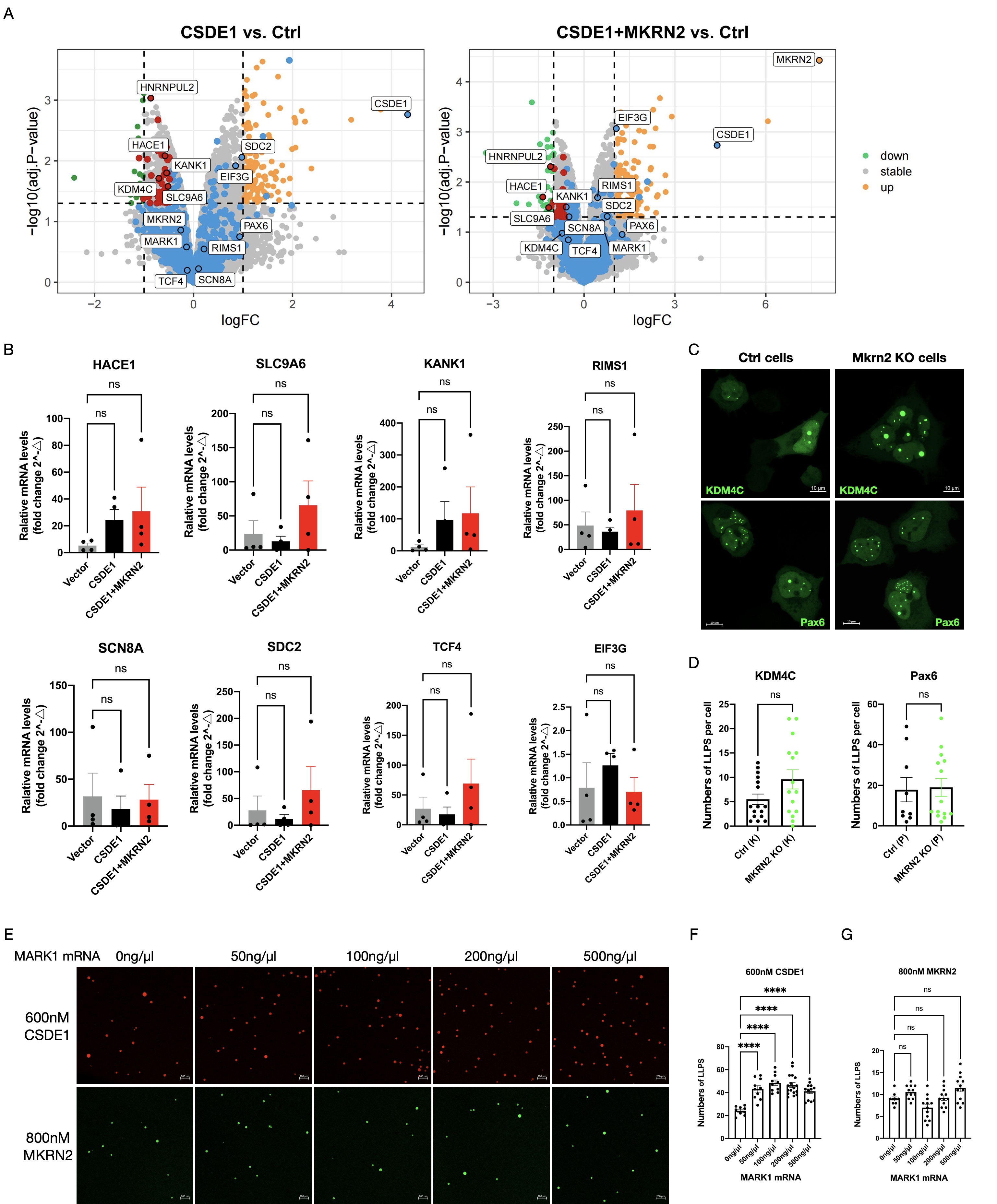


**Supplemental Figure 5.** ASD-relevant mRNAs induce LLPS of CSDE1 and MKRN2 in vitro. **(A)** Volcano plots for differentially expressed genes (DEGs). Left: CSDE1 vs Ctrl; Right: CSDE1+MKRN2 vs Ctrl. Green dots represent down-regulated genes while Orange dots represent upregulated genes. Blue dots represent SFARI overlapped genes. Red dots represent the core genes of SFARI overlapped genes after GESA enrichment analysis. The gray dashed lines indicate |log2(FoldChange)|> 1). (n = 3 pooled from Mkrn2 KO cells). **(B)** Analysis with RT-qPCR for the expression level of selected mRNAs in MKRN2 KO SH-SY5Y cells. **(C)** Expression of KDM4C and Pax6 mRNA in HEK293 Control and MKRN2 KO cells using MS2-MCP RNA reporter system. Images were acquired using the Zeiss LSM900 confocal microscope after 48h of transfection. Scale bar: 10 μm. **(D)** Quantification of KDM4C and Pax6 mRNA condensate number for data in (C).  show as means ± SEM, ns: not significant. **(E)** LLPS of purified recombinant CSDE1 or MKRN2 with MARK1 mRNAs in a gradient increasing concentration manner After 5min of induction, images were acquired using the Zeiss LSM900 confocal microscope. Scale bar: 200μm. **(F)** Quantification of CSDE1 condensate number for data in (E).  show as means ± SEM, ****P < 0.0001. **(G)** Quantification of MKRN2 condensate number for data in (E).  show as means ± SEM, ns: not significant.
