# Supplementary figures and images for: Liquid–liquid phase separation couples MKRN2-mediated ubiquitination of CSDE1 with neurodevelopmental disorders
Source: Front Cell Neurosci. 2026 Feb 11;20:1757304. doi: 10.3389/fncel.2026.1757304 (PMC12932526; doi:10.3389/fncel.2026.1757304)

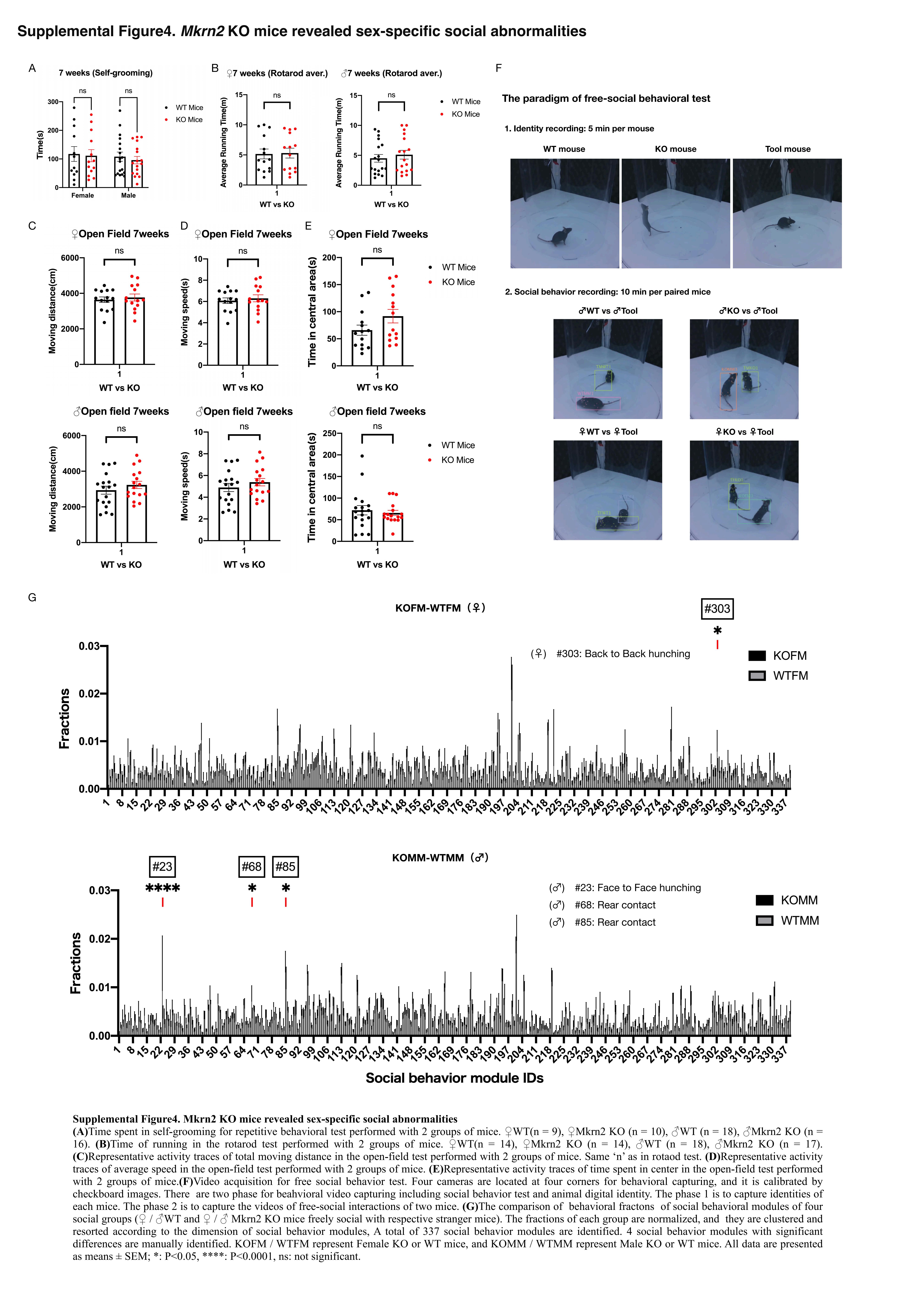

Supplement: Supplementary file 1 [file Data_Sheet_1.ZIP › Supplementary_Material-Zi Wang /Suppl. Fig.4.jpg]

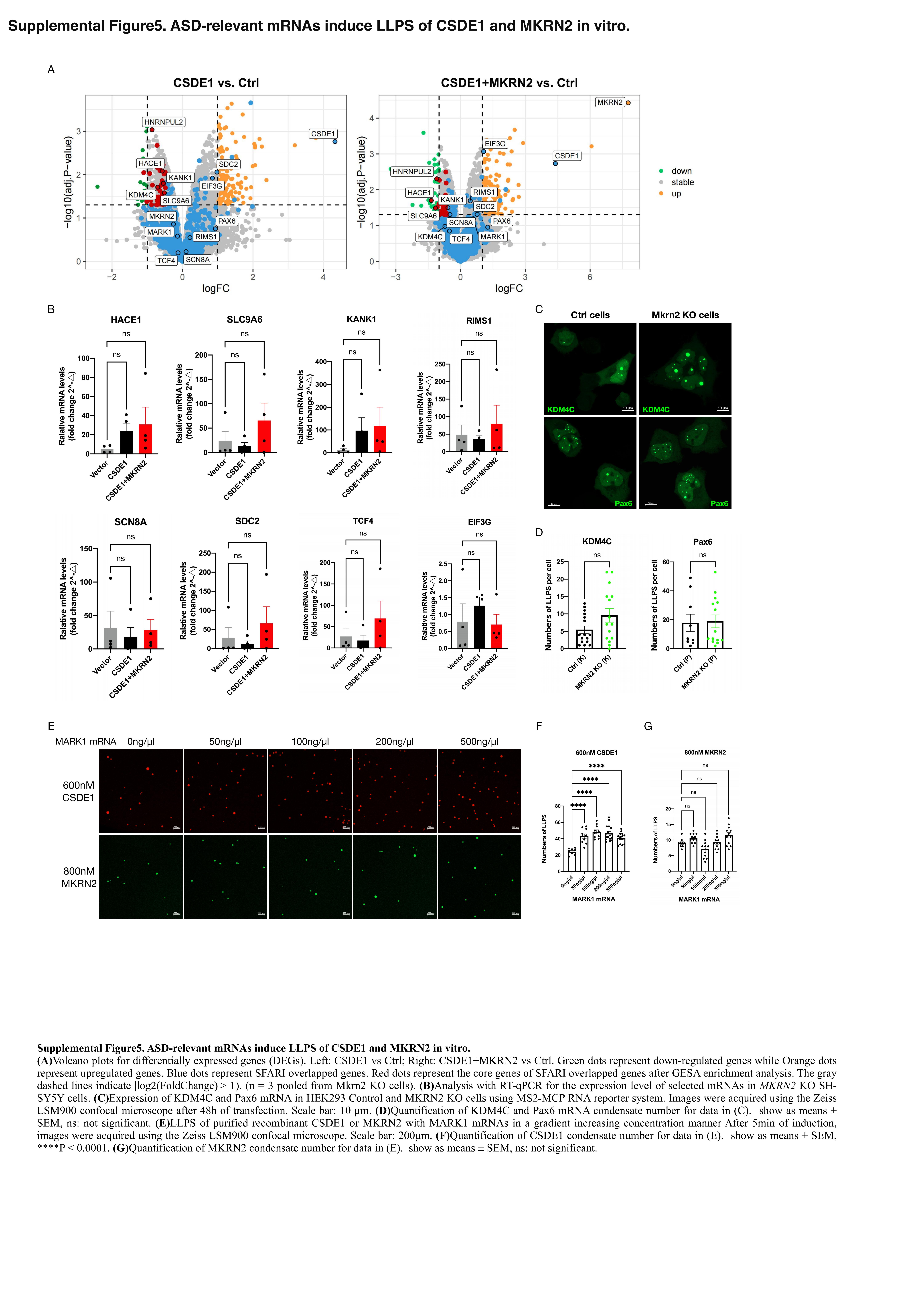

Supplement: Supplementary file 1 [file Data_Sheet_1.ZIP › Supplementary_Material-Zi Wang /Suppl. Fig.5.jpg]

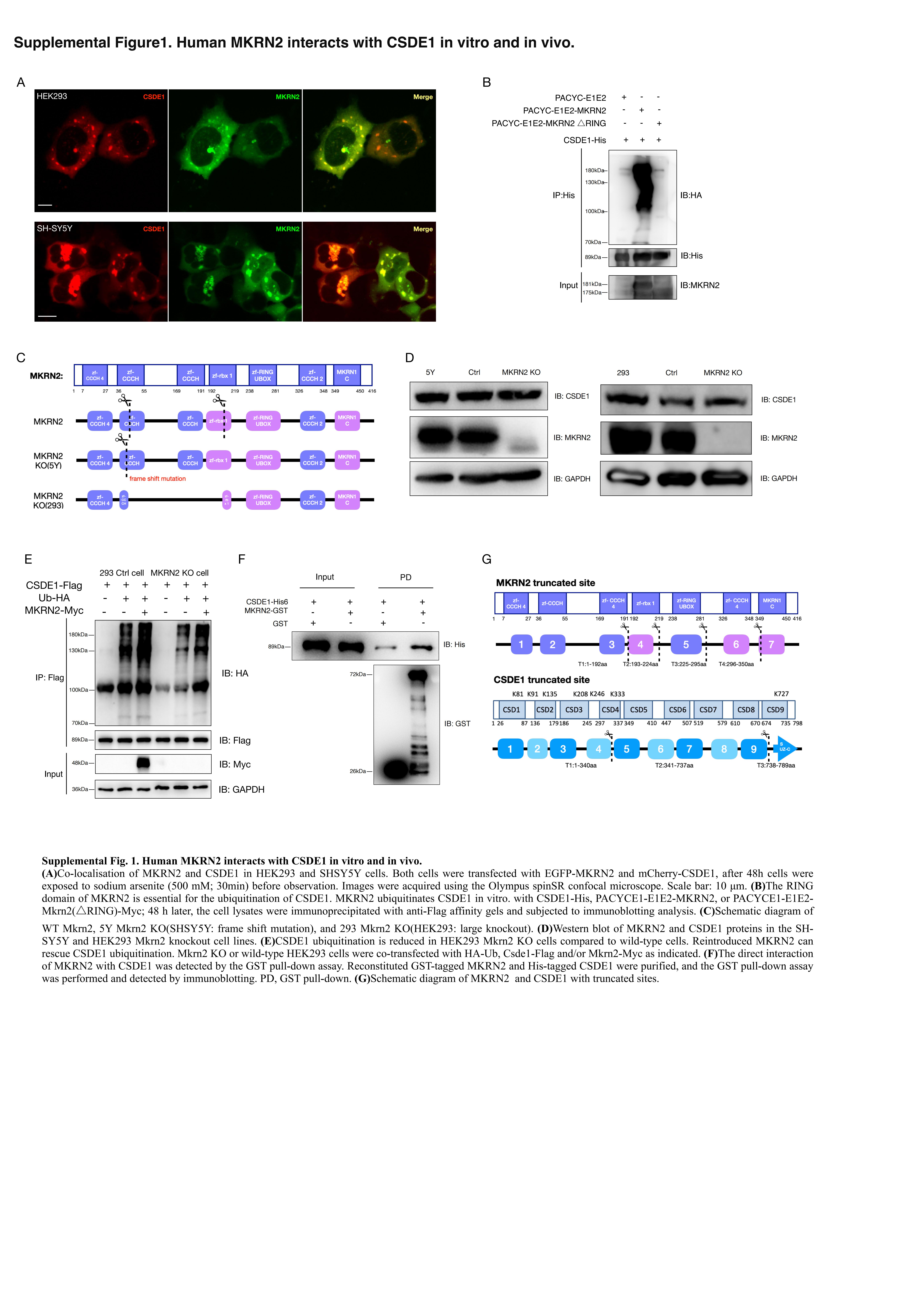

Supplement: Supplementary file 1 [file Data_Sheet_1.ZIP › Supplementary_Material-Zi Wang /Suppl. Fig.1.jpg]

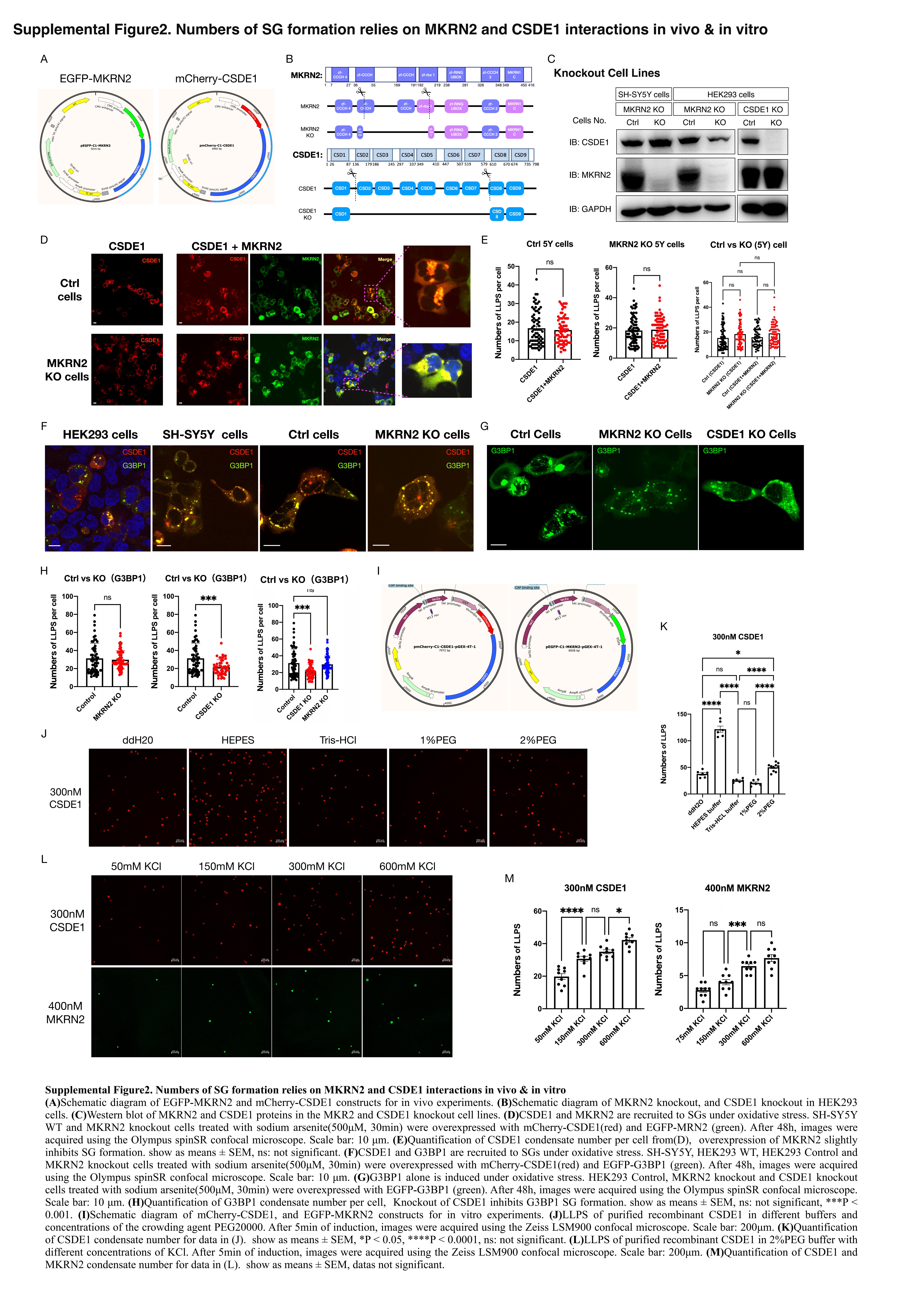

Supplement: Supplementary file 1 [file Data_Sheet_1.ZIP › Supplementary_Material-Zi Wang /Suppl. Fig.2.jpg]

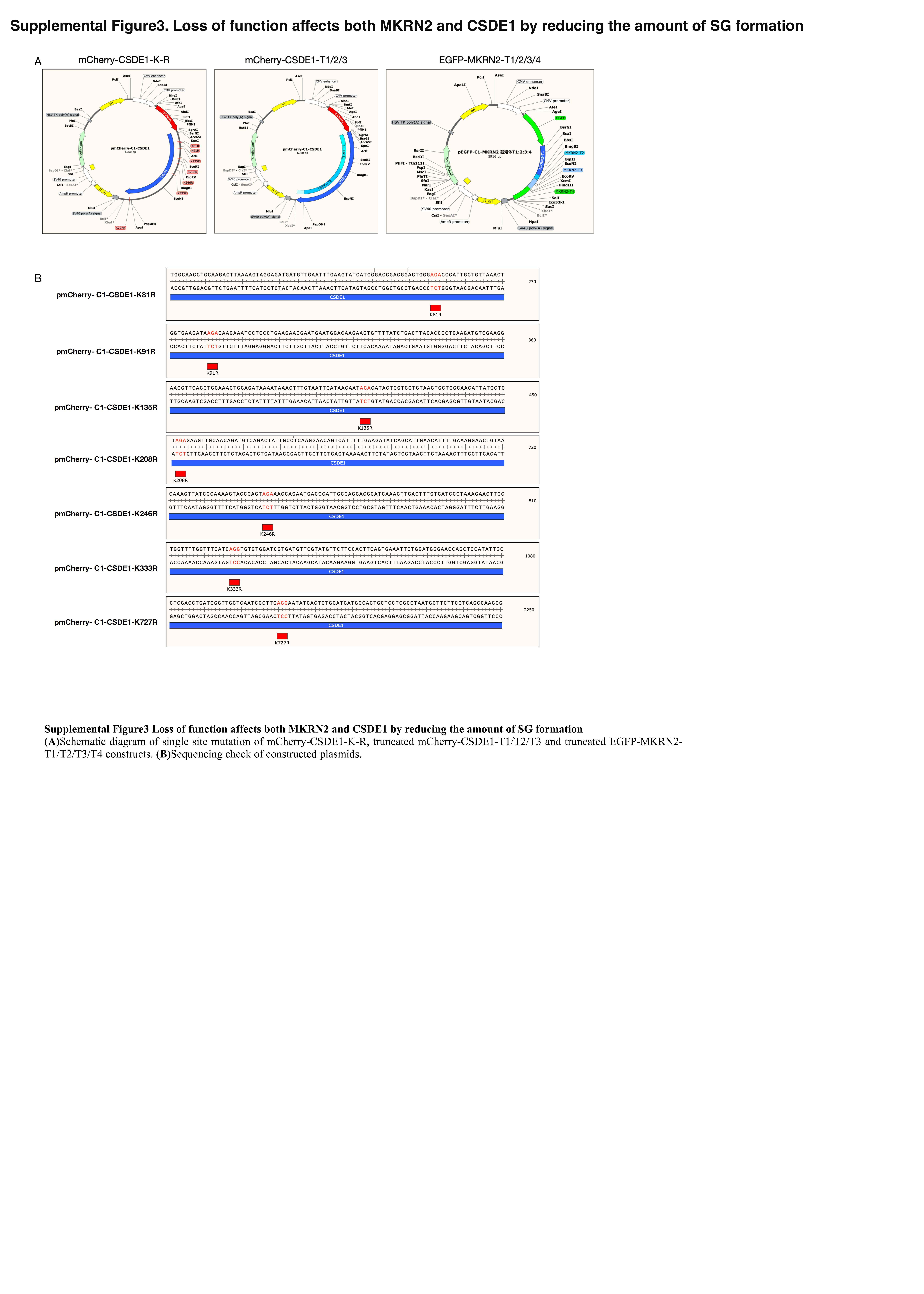

Supplement: Supplementary file 1 [file Data_Sheet_1.ZIP › Supplementary_Material-Zi Wang /Suppl. Fig.3.jpg]
